# Chilling stress response in tobacco seedlings: insights from transcriptome, proteome, and phosphoproteome analyses

**DOI:** 10.3389/fpls.2024.1390993

**Published:** 2024-05-28

**Authors:** Xiuhong Shao, Zhenchen Zhang, Faheng Yang, Yongchao Yu, Junjie Guo, Jiqin Li, Tingyu Xu, Xiaoying Pan

**Affiliations:** ^1^ Guangdong Key Laboratory for Crops Genetic Improvement, Crops Research Institute, Guangdong Academy of Agricultural Sciences (GAAS), Guangdong Provincial Engineering & Technology Research Center for Tobacco Breeding and Comprehensive Utilization, Guangzhou, China; ^2^ China National Tobacco Corporation, Guangdong Company, Guangzhou, China

**Keywords:** chilling stress, molecular mechanism, transcriptome, proteome, phosphoproteome, tobacco

## Abstract

Tobacco (*Nicotiana tabacum* L.) is an important industrial crop, which is sensitive to chilling stress. Tobacco seedlings that have been subjected to chilling stress readily flower early, which seriously affects the yield and quality of their leaves. Currently, there has been progress in elucidating the molecular mechanisms by which tobacco responds to chilling stress. However, little is known about the phosphorylation that is mediated by chilling. In this study, the transcriptome, proteome and phosphoproteome were analyzed to elucidate the mechanisms of the responses of tobacco shoot and root to chilling stress (4 °C for 24 h). A total of 6,113 differentially expressed genes (DEGs), 153 differentially expressed proteins (DEPs) and 345 differential phosphopeptides were identified in the shoot, and the corresponding numbers in the root were 6,394, 212 and 404, respectively. This study showed that the tobacco seedlings to 24 h of chilling stress primarily responded to this phenomenon by altering their levels of phosphopeptide abundance. Kyoto Encyclopedia of Genes and Genomes analyses revealed that starch and sucrose metabolism and endocytosis were the common pathways in the shoot and root at these levels. In addition, the differential phosphopeptide corresponding proteins were also significantly enriched in the pathways of photosynthesis-antenna proteins and carbon fixation in photosynthetic organisms in the shoot and arginine and proline metabolism, peroxisome and RNA transport in the root. These results suggest that phosphoproteins in these pathways play important roles in the response to chilling stress. Moreover, kinases and transcription factors (TFs) that respond to chilling at the levels of phosphorylation are also crucial for resistance to chilling in tobacco seedlings. The phosphorylation or dephosphorylation of kinases, such as CDPKs and RLKs; and TFs, including VIP1-like, ABI5-like protein 2, TCP7-like, WRKY 6-like, MYC2-like and CAMTA7 among others, may play essential roles in the transduction of tobacco chilling signal and the transcriptional regulation of the genes that respond to chilling stress. Taken together, these findings provide new insights into the molecular mechanisms and regulatory networks of the responses of tobacco to chilling stress.

## 1 Introduction

Chilling stress is a common natural stress that is encountered during the cultivation of crops, and it severely affects their growth, development, metabolism, spatial distribution and agricultural productivity (Chinnusamy et al., 2007; Zhu et al., 2007). Tobacco (*Nicotiana tabacum* L.) is an important industrial crop that originated from tropical and subtropical regions; it is a thermophilic crop that is sensitive to cold (Jin et al., 2017; Hu et al., 2018; Luo et al., 2022). Tobacco plants that have been subjected to chilling stress while they are seedlings can be severely damaged. Chilling stress can prolong the age of seedlings and delay the period for their transplantation. This delay can result in plants that are susceptible to high temperatures during the later stages of field growth and prone to vascular diseases, such as tobacco bacterial wilt (*Ralstonia solanacearum*). Importantly, since leaves are the parts of tobacco plants that are harvested and used industrially, chilling stress can advance the reproductive growth in plants, which induces the phenomenon of early flowering (Xu et al., 2022). The occurrence of early flowering decreases the yield and quality of tobacco leaves, which has serious economic effects on the areas that produce tobacco. The presence of these lower quality leaves substantially limits the ability of tobacco plants to produce the high-quality raw materials that the plants are grown for. The most economical and fundamental method to prevent and control the early flowering induced by low temperatures is to identify resistance genes and cultivate new cultivars of tobacco that can resist chilling stress and flower later.

Plants have developed complex and efficient mechanisms to resist and adapt to chilling stress over the course of long-term evolution, and they can adaptively adjust at the physiological, biochemical, cellular and molecular levels (Hu et al., 2022; Luo et al., 2022). In recent years, significant progress has been made in elucidating how plants perceive and transduce chilling signals (Penfield, 2008; Zhu, 2016; Wang et al., 2017; Shi et al., 2018; Ding et al., 2019; Mehrotra et al., 2020). It is a well-known model that the perception of temperatures can fluctuate. Such fluctuations in temperature decrease membrane fluidity and rearrange the cytoskeleton, which is followed by an influx of calcium (Ca^2+^) into the cytoplasm; this activates the responses that occur downstream (Guo et al., 2018; Tan et al., 2021). Among these downstream responses, ICE-CBF-COR is the pathway that has been studied the most of those that respond to chilling stress (Gilmour et al., 1998; Chinnusamy et al., 2007; Jia et al., 2016; Wang et al., 2017; Shi et al., 2018). This pathway is critical to the responses of plants to chilling stress. CBFs (C-repeat-binding factor), a class of transcription factors (TFs) (Gilmour et al., 1998), are regulated by ICE1 (inducer of CBF expression l) (Chinnusamy et al., 2003). They can activate the levels of expression of downstream genes, including *COR* (cold-regulated), *LTI* (low-temperature induced), and *RD* (responsive to dehydration) (Zhu et al., 2007), which thereby improves the ability of plants to tolerate low temperatures. Other TFs, such as NAC1 (Ma et al., 2013; Yin et al., 2024) and CAMTAs (Kim et al., 2013), and kinase genes, including *VaCPK20* (Dubrovina et al., 2015), *GsLRPK* (Yang et al., 2014), and the FERONIA receptor-like kinase gene *MdMRLK2* (Jing et al., 2023), are involved in this resistance to chilling stress. The development of omics technology enabled plant science researchers to reveal the complex mechanisms of the responses of plants to chilling stress. In recent years, substantial progress has been made in using transcriptomics, proteomics and metabolomics to elucidate the molecular mechanisms that tobacco plants use to tolerate chilling stress. Many genes have been identified at the transcriptomic level in tobacco plants that have been subjected to chilling treatment. These include the *COR* genes, *NbXTH22* (flower development-related gene) and TFs (*ABI3/VP1*, *ARR−B*, *WRKY*, *GRAS*, *AP2−EREBP* and *C2H2*) (Hu et al., 2016; Luo et al., 2022; Xu et al., 2022). The proteins involved in photosynthesis, signal transduction, carbon and energy metabolism, RNA processing, ROS scavenging, and protein synthesis and degradation are responsive to chilling stress at the proteomic level (Jin et al., 2011; Hu et al., 2018). In addition, at the transcriptomic and metabolic levels, the K326 cultivar of tobacco had much higher levels of gene expression and metabolites that accumulated compared with those in a cultivar that is sensitive to chilling stress (Jin et al., 2017). Several pathways, including starch and sucrose metabolism, flavonoid biosynthesis, and phenylpropanoid biosynthesis among others, were enriched by the integration of transcriptomic and metabolomic analyses. These techniques identified candidate genes, such as those for the chlorophyll a-b binding protein, ATPases, and UDP-glucosyltransferases among others (Hu et al., 2022). However, there has been extremely limited molecular characterization of the phosphoproteome that responds to chilling stress in tobacco. Post-translational modifications of the proteins, particularly phosphorylation, have been reported to play an important role in the adaptation of plants to chilling stress (Tan et al., 2021). Currently, phosphoproteome analyses have been applied in plants that included *Arabidopsis thaliana* (Kamal et al., 2020; Tan et al., 2021), tomato (*Solanum lycopersicum* L.) (Hsu et al., 2018), maize (*Zea mays* L.) (Xing et al., 2022), banana (*Musa* spp.) (Gao et al., 2017), and cucumber (*Cucumis sativus* L.) (Zhou et al., 2023). However, the overall characteristics of phosphorylation modifications in response to chilling stress in tobacco seedlings remain unknown.

Despite some progress in elucidating the molecular mechanisms used by tobacco to tolerate chilling stress, there is limited understanding of the post-translational phosphorylation modifications of proteins that are mediated by this type of stress. Currently, the comprehensive analysis of two or three types of omics data provides an important way to elucidate the regulatory mechanisms that plants use to respond to biotic or abiotic stress (Hu et al., 2022; Peng et al., 2023). Samples of tobacco seedlings were analyzed at the transcriptomic, proteomic and phosphoproteomic levels to determine the global alteration of genes and proteins and the levels of their phosphorylation from 24 h of chilling treatment. Additionally, unlike previous studies that primarily focused on tobacco leaves, the tobacco seedlings were divided into two parts of tissues for analysis, including its shoot (S) and root (R). The goal was to monitor the differences in the levels of expression of genes and the abundance of proteins and phosphopeptides between the shoot and root tissues after they had been subjected to chilling stress. This study will contribute to a better understanding of the regulatory network that tobacco seedlings use to respond to chilling stress.

## 2 Materials and methods

### 2.1 Plant growth and chilling treatment

*Nicotiana tabacum* L. cv. K326 was used in this study. K326 seeds were stored in the laboratory, and they were sown in plastic pots filled with a sterile mixture of peat culture substrate, carbonized chaff, and perlite (3:2:1, v/v/v). The seeds were germinated and cultured in a climate chamber (25 °C, 16 h/8 h light/dark, and 70% relative humidity) until the plants had reached the six-leaf stage. The seedlings were then transferred to another chamber for chilling treatment at 4 °C for 24 h, which replicated the treatments of previous studies (Jin et al., 2017; Hu et al., 2018). Seedlings cultured at 25 °C were used as the control. All the leaves and stems of each plant were mixed together as one shoot sample, and all the roots of each plant served as one root sample. Shoot and root samples were collected from the control and treated seedlings. The control and those treated with chilling stress were designated 0 h and 24 h, respectively. Three biological replicates of samples were immediately frozen in liquid nitrogen and stored at -80 °C for the subsequent extraction of RNA and proteins.

### 2.2 RNA extraction and transcriptome sequencing

The transcriptomes of 12 samples (two tissues × two time points × three biological replicates) were sequenced. The total RNA was extracted using the TRIzol reagent (Invitrogen, Carlsbad, CA, USA). An RNA-seq library was constructed, and clean reads were acquired and mapped to the reference genome as previously described (Pan et al., 2021). The transcriptome data were deposited in the SRA of the NCBI (accession number PRJNA1093408).

### 2.3 Protein extraction and digestion

The samples were ground individually in liquid nitrogen and lysed with SDT lysis buffer that contained SDS (4%, w/v), triethylammonium bicarbonate (TEAB, 10%, v/v), and 1M DTT (1%, v/v) and then ultrasonicated on ice for 5 min. The lysate was incubated at 95 °C for 8–15 min and then in an ice bath for 2 min before centrifugation at 12000 g for 15 min at 4 °C. The supernatant was alkylated with sufficient iodoacetamide (IAM) for 1 h in the dark at room temperature. The samples were completely mixed with 4-fold the volume of precooled acetone by vortexing and incubation at -20 °C for at least 2 h. The samples were then centrifuged at 12000 g for 15 min at 4 °C, and the precipitate was collected. It was washed with 1 mL of cold acetone, and the pellet was dissolved in Dissolution Buffer (DB buffer). A Bradford protein quantitative kit (Beyotime Biotech. Inc., Nanjing, China) was used to determine the concentration of proteins.

DB buffer (8 M urea and 100 mM TEAB, pH 8.5) was used to bring each sample of protein to a volume of 100 μL, and trypsin and 100 mM TEAB buffer were added. The sample was mixed and digested at 37°C for 4 h. Trypsin and calcium chloride (CaCl_2_) were then added and incubated overnight to digest the protein. The digested sample was mixed with formic acid, adjusted to pH < 3, and centrifuged at 12000 g for 5 min at room temperature. The supernatant was slowly loaded onto a C18 desalting column and washed three times with washing buffer (0.1% formic acid and 3% acetonitrile). Elution buffer (0.1% formic acid and 70% acetonitrile) was then added, and the eluents of each sample were collected and lyophilized.

### 2.4 Enrichment of the phosphopeptides

The phosphopeptides were enriched using Fe immobilized metal affinity chromatography (IMAC-Fe). The lyophilized powder was dissolved with binding buffer (No. OSFP0005; Shanghai Omicsolution Co., Ltd., Shanghai, China) and centrifuged at 12000 g for 5 min at 4°C. The IMAC-Fe column (Thermo Fisher Scientific, Waltham, MA, USA) was prepared with C18 column packing (Dr. Maisch HPLC GmbH, Ammerbuch, Germany), pre-column (2 cm × 75 μm, 3 μm), and analytical column (15 cm × 150 μm, 1.9 μm). The diameter of pre-column packing and analytical column packing was 3 μm and 1.9 μm, respectively, and the aperture was 120 Å. The supernatant was added to the pretreated IMAC-Fe column, incubated at room temperature for 30 min, and centrifuged at 2000 g for 30 s, and the column was washed once with washing solution and once with water. The sample was centrifuged again at 2000 g for 30 s, and the tube was discarded and replaced with a new, clean one. Elution buffer was then added. The polypeptide eluent was collected and lyophilized.

### 2.5 UPLC-MS/MS analysis

A nanoElute UPLC system (Bruker Daltonik, Bremen, Germany) coupled with a TIMS-TOF pro2 mass spectrometer (Bruker Daltonik) was used to perform ultra-high-performance liquid chromatography-tandem mass spectrometry (UPLC-MS/MS) analyses at Novogene Co., Ltd. (Beijing, China). First, mobile phases A (100% water and 0.1% formic acid) and B (80% acetonitrile and 0.1% formic acid) were prepared. The lyophilized powder was then dissolved in 10 μL of mobile phase A and centrifuged at 14000 g for 20 min at room temperature. A total of 200 ng of supernatant was used for detection. The nanoElute UPLC system coupled with the TIMS-TOF pro2 mass spectrometer was used to perform shotgun proteomics while operating in the data-dependent acquisition (DDA) mode. A linear gradient elution was used to separate the peptides in an analytical column (15 cm × 100 μm, 1.9 μm). The separated peptides were analyzed by TIMS-TOF pro2, with an ion source of Captive Spray and a spray voltage of 1.5 kV, by analyzing full scan ranges from m/z 100 to 1700. The Ramp time was 100 ms, and the Lock Duty Cycle was 100%. The PASEF settings were 10 MS/MS scans during 1.17 s; the cutoff of ion intensity was 2500, and the target of scheduling was 10000. The raw data detected by the MS were designated “.d”.

The search engine MaxQuant was used to search all the resulting spectra against the UniProt database. The search parameters of MaxQuant were established as follows: the mass tolerance for the precursor ion was 20 ppm, and the mass tolerance for the production was 0.05 Da. The immobilized modification was carbamidomethyl, while the oxidation of methionine and the addition of a phospho motif to serine (S), threonine (T) and tyrosine (Y) were the dynamic modifications. Acetyl, met-loss and met-loss+acetyl were modified at the N terminus, and a maximum of two missed cleavage sites were allowed. The quality of analytic results was improved by using MaxQuant to more thoroughly filter the retrieval results. Peptide Spectrum Matches (PSMs) with a credibility > 99% were identified as credible PSMs. A credible protein was defined as a protein that contained at least one unique peptide. The credible PSMs and proteins were retained and implemented with a false discovery rate (FDR) of ≤ 1.0%, and a *t*-test was used to analyze the quantitative results of the proteins. The proteins whose quantification differed significantly between the experimental and control groups with *p* < 0.05 and FC > 1.5 or FC < 0.67 (fold-change, FC) were defined as differentially expressed proteins (DEPs).

### 2.6 Bioinformatics analysis

*N. tabacum* Ntab-TN90 was used as the reference genome (Sierro et al., 2014). The data of three omics were analyzed by Novogene Co., Ltd. (Beijing, China). Transcripts with an adjusted *p*-value (padj) < 0.05 and |log_2_FC| > 1 were identified as differentially expressed genes (DEGs). Differential phosphopeptides were filtered for an average FC of > 1.5 or < 0.67 (P < 0.05). A principal components analysis (PCA) was conducted using the PCAtools packages in the R environment (Xing et al., 2022). Heatmaps of the Pearson correlation, Venn diagrams and volcano plots were created with ggplot2 (Wickham, 2016). Heatmaps of the significantly different phosphorylation sites were generated with the pheatmap R packages. The Gene Ontology (GO) and KEGG pathway enrichments were analyzed using the clusterProfiler R package with P < 0.05. A motif analysis was conducted using Wagih’s method, and the ± 7 residues that surrounded the chilling responsive phosphorylation sites with an occurrence threshold > 20 and P < 10^−6^ were enriched (Schwartz and Gygi, 2005; Wagih et al., 2016). The StringDB Protein Interaction Database (http://string-db.org/) was used to analyze the interactions of differential phosphoproteins with a high confidence (score > 0.7). Cytoscape software was then used to construct the protein–protein interaction (PPI) network.

### 2.7 Analysis of gene expression

The total RNA obtained in Section 2.2 was reverse-transcribed using a PrimeScript™ RT reagent Kit with gDNA Eraser (Perfect Real Time; Takara Bio, Shiga, Japan) (Pan et al., 2021). Five DEGs in the shoot and root that had been subjected to chilling were randomly selected for validation by real-time quantitative reverse transcription PCR (qRT-PCR) using the primers listed in **Supplementary Table S1**. The 2^−ΔΔCt^ method was used to calculate the relative levels of gene expression. The *actin* gene was used as the internal control to normalize the levels of gene expression (Jain et al., 2006). Microsoft Excel 2019 (Redmond, WA, USA) was used to generate figures of the levels of relative expression. A Student’s *t*-test was used to analyze the significance between samples.

## 3 Results

### 3.1 Proteomic profiles of the tobacco seedlings in response to chilling stress

**Figure 1:**
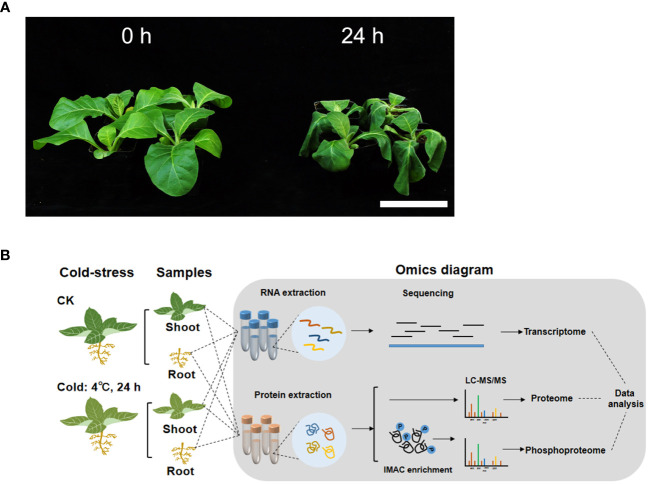
The phenotype of tobacco seedlings and workflow for the transcriptome, proteome and phosphoproteome profiling under chilling stress. **(A)** Phenotype of tobacco seedlings under chilling stress. K326 seedlings at the six-leaf stage were treated at 4 °C for 24 h (24 h), and the seedlings before chilling treatment (0 h) were used as the control. Scale bar=10 cm. **(B)** Workflow for the transcriptome, proteome and phosphoproteome profiling of tobacco seedlings under chilling stress.

**Figure 2:**
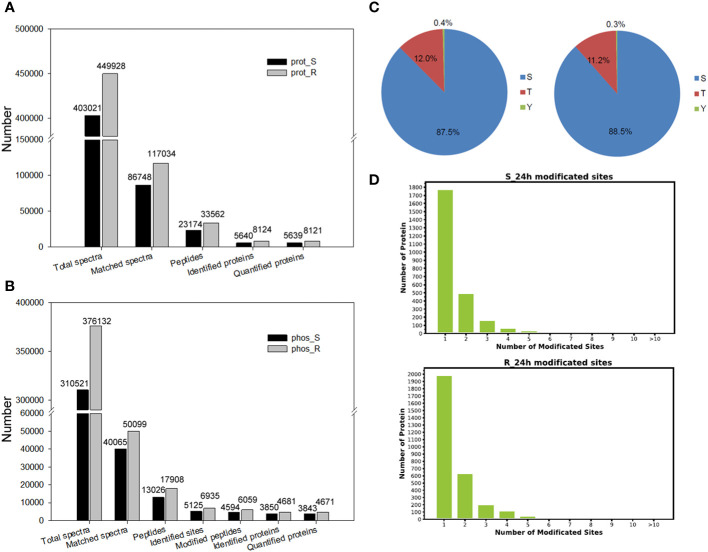
Proteome and phosphoproteome analyses of tobacco in response to chilling stress. **(A)** Proteomic identification data of tobacco under chilling stress. Prot, the proteome. S, shoot data. R, root data. **(B)** Phosphoproteomic identification data of tobacco under chilling stress. Phos, the phosphoproteome. **(C)** Proportion of the Ser, Thy and Tyr sites in phosphoproteins. Left pie chart, shoot data. Right pie chart, root data. **(D)** The number of phosphorylation sites in proteins.

**Figure 3:**
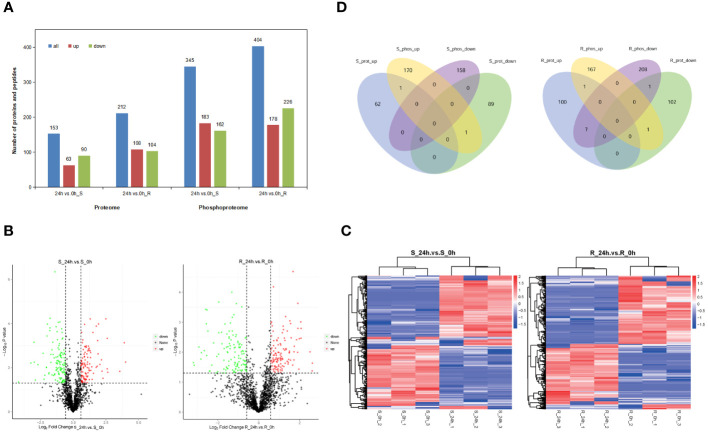
Analyses of the DEPs and differential phosphopeptide data and significantly different phosphorylation sites in response to chilling stress. **(A)** DEPs and differential phosphopeptides in the proteome and phosphoproteome, respectively. Proteins and phosphopeptides that significantly differed quantitatively between the experimental and control groups were defined as DEPs and differential phosphopeptides (P < 0.05, FC > 1.5 or FC < 0.67. **(B)** Volcano plots of quantified phosphorylation sites. **(C)** Heatmaps of significantly different phosphorylation sites. The phosphorylation sites were filtered for an average FC of > 1.5 or < 0.67 with P < 0.05. **(D)** Venn diagram of the DEPs and corresponding proteins of the differential phosphopeptides in the proteome and phosphoproteome.

The proteome of both the shoot and root tissues was analyzed to study the response of tobacco seedlings to chilling stress at the protein levels (**Figure 1A**). A simple workflow of the proteomic analysis under chilling stress is shown in **Figure 1B**. A PCA was performed on samples of the shoot and root proteomes, and the two sample groups of proteomic data could be separated basically (**Supplementary Figure S1**). A total of 403,021 secondary spectra were obtained in the shoot proteome, and 86,748 were matched spectra; 23,174 peptides and 5,640 proteins were identified with 5,639 of them quantifiable (**Figure 2A** **;** **Supplementary Table S2**). A total of 449,928 secondary spectra were obtained from the root proteome, and 117,034 were matched spectra; 33,562 peptides and 8,124 proteins were identified with 8,121 of them quantifiable (**Figure 2A** **;** **Supplementary Table S2**). These results showed that fewer proteins were quantified in the shoot than in the root. In addition, the DEPs in the shoot and root of tobacco seedlings under chilling stress were examined. Compared with 0 h, there were 153 DEPs in the shoot after 24 h of chilling treatment. A total of 63 increased significantly, while 90 decreased. There were 212 DEPs in the root, including 108 that significantly increased and 104 that decreased (**Figure 3A**).

**Figure 4:**
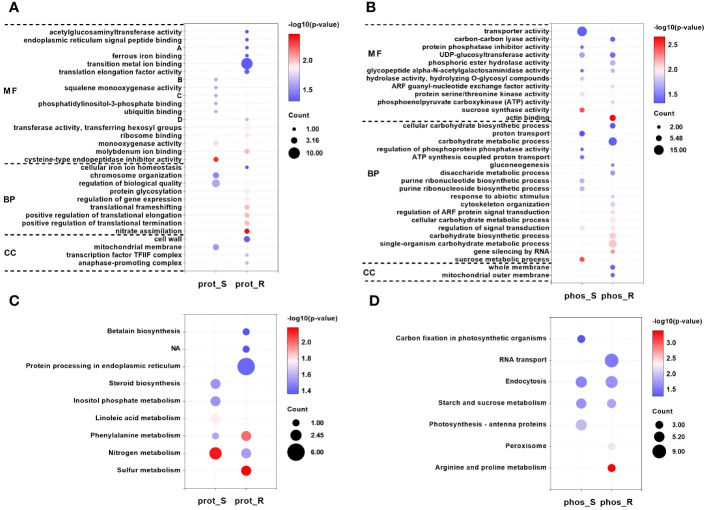
GO and KEGG enrichment analyses of DEPs and corresponding proteins of the differential phosphopeptides in response to chilling stress. GO **(A)** and KEGG **(C)** enrichment analyses of DEPs in the proteome. **(A)**, UDP-glucose: glycoprotein glucosyltransferase activity; **(B)**, 2,3-bisphosphoglycerate-independent phosphoglycerate mutase activity; **(C)**, 2-C-methyl-D-erythritol -2,4-cyclodiphosphate synthase activity; **(D)**, 2-amino-4-hydroxy-6-hydroxymethyldihydropteridine diphosphokinase activity. GO **(B)** and KEGG **(D)** enrichment analyses of corresponding proteins of the differential phosphopeptides in the phosphoproteome. GO and KEGG enrichment analyses were performed with P < 0.05 as the threshold.

A GO enrichment analysis was then performed on the 153 DEPs in the shoot, and seven molecular function (MF), two biological process (BP) and one cellular component (CC) terms were significantly enriched (**Figure 4A** **;** **Supplementary Table S3**). Moreover, the results of 212 DEPs in the root were different from those in the shoot, and there were more enriched terms for the proteins that responded to chilling, including 10 MF, seven BP, and three CC (**Figure 4A** **;** **Supplementary Table S3**). In addition, the GO terms that were shared between the shoot and root were compared, and no common GO term was identified between the two tissues. Moreover, the results of a KEGG enrichment analysis on the 153 DEPs in the shoot showed that five pathways were significantly enriched, including nitrogen metabolism, linoleic acid metabolism, phenylalanine metabolism, steroid biosynthesis and inositol phosphate metabolism (**Figure 4C** **;** **Supplementary Table S4**). Moreover, six pathways were significantly enriched for the 212 DEPs in the root, including sulfur metabolism, phenylalanine metabolism, nitrogen metabolism, protein processing in endoplasmic reticulum, and betalain biosynthesis among others (**Figure 4C** **;** **Supplementary Table S4**). These results showed that nitrogen metabolism and phenylalanine metabolism were the common pathways between the two tissues.

In addition, the kinases and TFs in the proteome that responded to chilling stress were analyzed. At the levels of protein abundance, three and four kinases in the shoot and root were found to respond to chilling treatment, respectively (**Supplementary Table S5**). Among them, one kinase increased, and two kinases decreased in the shoot. In contrast, three kinases increased in the root, including leucine-rich repeat receptor-like protein kinase PXC1, LRR receptor-like serine/threonine-protein kinase RPK2 and leucine-rich repeat receptor-like serine/threonine-protein kinase BAM1. However, one serine/threonine-protein kinase 4-like decreased. In addition, five TFs in the root responded to chilling treatment, and two and three TFs increased and decreased, respectively (**Supplementary Table S5**). No changes in the shoot were detected at the levels of protein abundance.

### 3.2 Chilling-responsive proteins related to the pathways of phenylalanine and nitrogen metabolism in tobacco seedlings

The differential proteins were extracted from the proteome to identify proteins in the significantly enriched pathways in both the shoot and root of tobacco that had been subjected to chilling stress. The DEPs enrichment data of the proteome indicated that there were two pathways in both the shoot and root of the tobacco seedlings, including phenylalanine metabolism and nitrogen metabolism. Three DEPs related to phenylalanine metabolism were identified, including one in the shoot and two in the root; they were all phenylalanine ammonia-lyase (PAL) and increased significantly at the levels of protein abundance (**Supplementary Table S6**). PAL enzymes are involved in plant growth and development, stress resistance and the biosynthesis of secondary metabolites. Studies have shown that the activity of PAL increased in wheat (*Triticum aestivum* L.) (Kolupaev et al., 2018), tobacco (Gu et al., 2021) and tomato (Pappi et al., 2021) under chilling stress.

The early chilling stress responses of tobacco seedlings (treated at 4 °C for 4 h) were also shown to affect nitrogen metabolism (Jin et al., 2011). In this study, five DEPs related to nitrogen metabolism were identified, and there were three in the shoot and two in the root; they were carbonic anhydrase (CA), nitrate reductase (NR) and cyanate hydratase (**Supplementary Table S6**). CA increased significantly in both the shoot and root, and there was a higher ratio of upregulation in the root (FC = 6.94), while NR decreased significantly in both tissues. Cyanate hydratase only decreased in the shoot at the levels of protein abundance. CA is an enzyme that catalyzes the conversion between bicarbonate (HCO_3_^-^) and carbon dioxide (CO_2_), and it is an important factor that determines the efficiency of photosynthetic carbon fixation. Similarly, it was upregulated in tobacco seedlings under short-term (4 h) chilling treatment (Jin et al., 2011). NR is the first key enzyme in the pathway of plant nitrogen metabolism, and it reduces nitrate (NO_3_^-^) to nitrite (NO_2_^-^). It was much less active in the shoot and root of rice (*Oryza sativa* L.) at 15 °C (Liu et al., 2013).

### 3.3 Analysis of the phosphoproteomic profiles and comparative analyses of DEPs and differential phosphopeptide corresponding proteins of the tobacco seedlings in response to chilling stress

The responses of tobacco seedlings to chilling stress at the phosphorylation levels were studied by analyzing the phosphoproteomes of both the shoot and root. The workflow of the phosphoproteomic analysis under chilling stress is shown in **Figure 1B**. The samples of the shoot and root phosphoproteome were analyzed using a PCA, and it was shown to clearly and sequentially separate the two sample groups of phosphoproteomic data (**Supplementary Figure S2**). A total of 13,026 peptides, 5,125 phosphorylation sites (≥ 0.75), 4,594 phosphopeptides (≥ 0.75) and 3,850 proteins were identified in the shoot phosphoproteome, and 3,843 were quantifiable (**Figure 2B** **;** **Supplementary Table S7**). A total of 17,908 peptides, 6,935 phosphorylation sites (≥ 0.75), 6,059 phosphopeptides (≥ 0.75) and 4,681 proteins were identified, and 4,671 were quantifiable in the root phosphoproteome (**Figure 2B** **;** **Supplementary Table S7**). Among the 5,125 phosphorylation sites identified in the shoot, 4,486 (87.5%) were from phosphoserine; 617 (12.0%) were found at threonine residues, and 22 (0.4%) were found at tyrosine residues (**Figure 2C**). Out of the 6,935 phosphorylation sites identified in the root, 6,136 (88.5%) were from phosphoserine, and 778 (11.2%) and 21 (0.3%) were found at threonine and tyrosine residues, respectively (**Figure 2C**). These results are consistent with the findings of previous studies in other plants (Tan et al., 2021; Xing et al., 2022; Zhou et al., 2023), which showed that the modifications of phosphorylation primarily occur on serine and threonine residues. In addition, the corresponding protein-phosphorylation site mapping revealed that the proteins with one phosphorylation site were the most abundant, followed by those with two phosphorylation sites (**Figure 2D**).

In addition, the differential phosphopeptides in the shoot and root of the tobacco seedlings were examined under chilling stress. The shoot phosphoproteome had 345 differential phosphopeptides; 183 increased significantly, and 162 decreased (**Figure 3A**). The root phosphoproteome revealed 404 differential phosphopeptides, including 178 that increased significantly and 226 that decreased (**Figure 3A**). In addition, the phosphorylation sites that were identified in the shoot and root were analyzed. There were 1,639 common phosphorylation sites in the shoot between the 24 h and 0 h samples. A total of 377 had changed significantly in the shoot; 199 increased significantly, and 178 decreased (**Figures 3B, C**). There were 1,929 common phosphorylation sites in the root with 445 that changed significantly; 201 of them increased significantly, and 244 decreased (**Figures 3B, C**). It was apparent that there were more differential phosphorylation sites in the root than in the shoot.

A GO analysis was then performed on the 330 corresponding proteins of the differential phosphopeptides in the shoot, and seven GO-MF terms were significantly enriched (**Figure 4B** **;** **Supplementary Table S8**), including sucrose synthase activity, protein serine/threonine kinase activity, and UDP-glucosyltransferase activity among others. In addition, there were seven GO-BP terms that were significantly enriched, including the sucrose metabolic process, regulation of signal transduction, and purine ribonucleotide biosynthetic process among others. Moreover, the results of the GO analysis for the 385 corresponding proteins of the differential phosphopeptides in the root showed that in contrast to the shoot, there were more enriched terms for the phosphoproteins that responded to chilling, including seven MF, 12 BP, and two CC (**Figure 4B** **;** **Supplementary Table S8**). In addition, the GO terms that were shared between the shoot and root were compared. They revealed that the tissues shared two MF terms and one BP. The GO-MF terms UDP-glucosyltransferase activity and glycopeptide α-N-acetylgalactosaminidase activity were enriched, and the GO-BP term regulation of signal transduction was enriched. Furthermore, the KEGG analysis for the 330 corresponding proteins of the differential phosphopeptides in the shoot showed that four pathways were significantly enriched, including photosynthesis-antenna proteins, starch and sucrose metabolism, and endocytosis and carbon fixation in photosynthetic organisms (**Figure 4D** **;** **Supplementary Table S9**). Moreover, the results of 385 corresponding proteins of the differential phosphopeptides in the root showed that five pathways were significantly enriched, including arginine and proline metabolism, peroxisome, starch and sucrose metabolism, endocytosis and RNA transport (**Figure 4D** **;** **Supplementary Table S9**). These results showed that starch and sucrose metabolism and endocytosis were the common pathways between the two tissues. This suggests that these pathways may play important roles in the responses of tobacco seedlings to chilling stress.

Moreover, a Venn analysis was performed on the DEPs and corresponding proteins of the differential phosphopeptides in the shoot and root to study the correlation between the proteome and phosphoproteome. There was only one common protein that increased between the proteome and phosphoproteome in the shoot, whereas no common protein that decreased was identified in the shoot (**Figure 3D**). Similarly, there was only one consensus protein that increased and decreased between the proteome and phosphoproteome in the root, respectively. There were seven proteins that increased in the proteome and decreased in the phosphoproteome (**Figure 3D**). These results showed that very few significantly changed proteins were shared by the proteome and phosphoproteome, which indicated that there was a lower correlation between the two omics.

### 3.4 Motif analysis of the chilling-responsive phosphopeptides and construction of the chilling-responsive phosphoprotein and phosphoprotein interaction networks

The kinases that were associated with the motifs that flank the phosphorylation sites that respond to chilling were predicted by their identification in the shoot and root. After 24 h of chilling treatment, 24 and 33 motifs were identified in the shoot and root, respectively (**Supplementary Figures S3A, B**). Among them, 23 motifs were centered on phosphoserine and one motif on phosphothreonine in the shoot, while 31 motifs were centered on phosphoserine and two motifs on phosphothreonine in the root. No motif was found to be centered on phosphotyrosine. The abundance of these motifs varied. The motifs with the highest ratios of the shoot and root were [sP] and [tP], which were pSer and pThr motifs, respectively, with 444 and 176 phosphopeptides in the shoot and 446 and 161 phosphopeptides in the root, respectively (**Supplementary Figures S3C, D** **;** **Supplementary Table S10**). This is consistent with previous studies in which the highest number of motifs were [sP] and [tP] (van Wijk et al., 2014; Hsu et al., 2018; Kamal et al., 2020). In addition to [sP], pSer motifs that were highly abundant also included [Rxxs], with 252 and 317 phosphopeptides in the shoot and root, respectively (**Supplementary Figures S3C ,D** **;** **Supplementary Table S10**). The motifs with the lowest ratios of the shoot and root were [LxKxxs] and [LxxSxs], respectively, which were all pSer motifs, with 51 and 50 phosphopeptides, respectively (**Supplementary Figures S3C, D** **;** **Supplementary Table S10**). Among the motifs with higher scores (> 300), 15 were the same in both the shoot and root. However, there were 5 and 13 in the shoot and root for the different motifs, respectively (**Supplementary Table S10**).

Next, the String database was used to analyze the interaction of differential phosphoproteins that responded to chilling stress in the shoot and root, and the PPI networks were then constructed using Cytoscape software. The interaction information of 39 and 40 differential phosphoproteins was obtained from the shoot and root, respectively, using confidence > 0.7 as the threshold. A0A1S4DPT4 (40S ribosomal protein S29), A0A1S4BXT5 (40S ribosomal protein S5-like), A0A1S3YAU9 (Chlorophyll a-b binding protein), A0A1S4AKK6 (Chlorophyll a-b binding protein), A0A1S3ZEB9 (60S acidic ribosomal protein P0), and Q0PWS5 (Chlorophyll a-b binding protein) among others were identified as hubs in the shoot in response to the chilling stress PPI network, which connected with six to 10 nodes (**Supplementary Figure S4A**). A0A1S3YSD8 (eukaryotic initiation factor 4A-3-like), A0A1S3ZMA0 (ribosome biogenesis protein BMS1 homolog), A0A1S3XC39 (protein LEO1 homolog isoform X1), and A0A1S3ZWJ5 (transcription factor IWS1-like) among others were identified as hubs in the root, which were connected by three to four nodes (**Supplementary Figure S4B**).

### 3.5 Identification of the chilling-responsive kinases and TFs in the phosphoproteome

The differential kinases and TFs were extracted from the phosphoproteome to study whether chilling stress plays an important role in the regulation of kinases and TFs. There were 23 and 31 kinases and nine and five TFs in the shoot and root, respectively (**Supplementary Table S5**). A total of 16 kinases increased in the shoot at the levels of phosphopeptide abundance, including three calcium-dependent protein kinases (CDPKs), two receptor-like protein kinases (RLKs), cold-responsive protein kinase 1 (CRPK1), CDPK-related kinase, and serine/threonine protein kinase IREH1 among others. Seven kinases decreased. These included two serine/threonine-protein kinase STN7 and two serine/threonine-protein kinase BLUS1-like among others (**Supplementary Table S5**). Moreover, 14 kinases increased in the root, including three RLKs, two CDPKs, two serine/threonine-protein kinase HT1-like, casein kinase, and chitin elicitor receptor kinase among others. A total of 17 kinases decreased, including four RLKs, two phosphoenolpyruvate carboxykinases, two serine/threonine-protein kinase HT1-like, and mitogen-activated protein kinase (MAPK) among others (**Supplementary Table S5**).

Five TFs increased in the shoot, including VIP1-like, TCP7-like, Abscisic acid-insensitive 5 (ABI5)-like protein 2, and plastid transcriptionally active 16 (PTAC16) among others, while four TFs decreased that included transcription initiation factor TFIID subunit 10-like, TCP2-like, IWS1-like and plastid transcriptionally active 10 (PTAC10)-like (**Supplementary Table S5**). Furthermore, four TFs increased in the root, including transcription initiation factor TFIID subunit 1-like, WRKY 6-like, IWS1-like and MYC2-like, while calmodulin-binding transcription activator 7 (CAMTA7) decreased (**Supplementary Table S5**). Taken together, the kinases and TFs of the tobacco seedlings that responded to chilling stress were primarily shown to respond at the levels of phosphopeptide abundance compared with the data from the proteome.

### 3.6 Chilling-responsive phosphoproteins related to the starch and sucrose metabolism and endocytosis pathways in tobacco seedlings

The differential phosphopeptide corresponding proteins were extracted from the phosphoproteome to identify phosphoproteins in the significantly enriched pathways in both the shoot and root of tobacco that had been subjected to chilling stress. In this study, two pathways were significantly enriched in both the shoot and root of tobacco seedlings, including starch and sucrose metabolism and endocytosis. Nine differential phosphoproteins related to the starch and sucrose metabolism were identified, including five in the shoot and four in the root. They were β-amylase (BAM), sucrose phosphate synthase (SPS), sucrose synthase (SuSy), and α,α-trehalose-phosphate synthase (**Supplementary Table S6**). In addition, BAM increased significantly in the shoot. The other three enzymes decreased in the shoot or root at the levels of phosphopeptide abundance. The activities of these enzymes have been reported to be related to chilling stress. First, BAM is an important starch hydrolase that hydrolyzes starch to β-maltose, which is quickly converted to glucose for the biosynthesis of sucrose. The overexpression of *BAM1* results in the increased activity of BAM, contents of soluble sugars, and chilling tolerance of the plant (Liang et al., 2023). Secondly, SPS and SuSy are enzymes that are intimately involved in the biosynthesis of sucrose, and their activities and the contents of sucrose increased after chilling treatment (Zhang et al., 2019). Third, trehalose-phosphate synthase (TPS) is involved in the biosynthesis of trehalose. The content of trehalose increased in banana under chilling stress (Gao et al., 2017). Studies have shown that phosphorylation can change the activity of enzymes, such as SPS, SuSy and TPS. For example, rice OsCPK17 can phosphorylate OsSPS4, which results in a decrease in the activity of OsSPS4 during the early stages of chilling stress (Almadanim et al., 2017). SuSy was phosphorylated by CDPK in tomato fruits (Anguenot et al., 2006), and its activity was altered by phosphorylation in broad bean (*Vicia faba* L.) (Polit and Ciereszko, 2012). The TPS was phosphorylated and decreased in the root of the tea plant (*Camellia sinensis* L.) that had been treated with glycine; this enhanced its activity (Li et al., 2023a). Taken together, this led to the hypothesis that under chilling stress, the downregulation of the abundance of phosphorylated SPS, SuSy and TPS in tobacco facilitated the accumulation of soluble sugars, such as sucrose and trehalose, thereby improving its chilling tolerance.

There were 13 differential phosphoproteins related to the endocytosis pathway (**Supplementary Table S6**), including six in the shoot and seven in the root. Among them, four increased significantly in the shoot or root at the levels of phosphopeptide abundance. The differential phosphoproteins included ADP-ribosylation factor GTPase-activating protein AGD5, clathrin interactor EPSIN 1-like, clathrin interactor EPSIN 2-like, actin cytoskeleton-regulatory complex protein PAN1-like, and brefeldin A-inhibited guanine nucleotide-exchange protein 2-like among others. This pathway was also one of the early pathways in *A. thaliana* and maize that responded to chilling stress (Tan et al., 2021; Xing et al., 2022). Endocytosis is a primary route for extracellular molecules and membrane proteins among others to enter the cells. It is required for many cellular processes, such as the delivery of nutrients, signaling transduction, and defense against pathogens (Chen et al., 2011; Fan et al., 2015). In plants, the major endocytic mechanism is clathrin-mediated endocytosis (CME) (Chen et al., 2011; Narasimhan et al., 2020), which requires many endocytic components, including AP2, EPSIN-like proteins, ADP-ribosylation factor GTPase-activating protein, and PAN1 among others (Naramoto et al., 2010; Chen et al., 2011; McMahon and Boucrot, 2011; Fan et al., 2015; Mettlen et al., 2018; Miao et al., 2018). The dephosphorylation of many endocytic components triggers the initiation of CME (Marks and McMahon, 1998; Tan et al., 2003; McMahon and Boucrot, 2011). In conclusion, these data led to the hypothesis that the phosphorylation or dephosphorylation of endocytic proteins in this study plays an important role in maintaining the stability of endocytosis mechanism, taking up transmembrane proteins and transducing chilling signals in response to chilling stress.

### 3.7 Chilling-responsive “photosynthesis-antenna proteins” and “carbon fixation in photosynthetic organisms” phosphoproteins in the shoot

Phosphoproteins in the significantly enriched pathways in the shoot of tobacco under chilling stress were identified by extracting the differential phosphopeptide corresponding proteins from the phosphoproteome. There were six differential phosphoproteins related to the “photosynthesis-antenna proteins” pathway in the shoot, and they were all chlorophyll a-b binding proteins (**Supplementary Table S6**). Strikingly, they unanimously increased at the levels of phosphopeptide abundance, indicating that the phosphorylation of these proteins plays important roles in the response to chilling stress in the shoot. A similar result was identified as one of 12 key candidate genes in the response of tobacco to chilling stress (Hu et al., 2022). The phosphoproteins related to the photosynthetic pathway in cucumber were also upregulated under chilling stress (Zhou et al., 2023). Three differential phosphoproteins related to the “carbon fixation in photosynthetic organisms” pathway were identified, and they were all glyceraldehyde-3-phosphate dehydrogenases (GAPDH) (**Supplementary Table S6**). Notably, they also all increased in the shoot at the levels of phosphopeptide abundance, which indicated that the phosphorylation of these proteins may play important roles in the response to chilling stress. GAPDH is a highly conserved protein in all organisms that plays a core role in the carbon metabolism of cells, and it is one of the most basic enzymes that maintains the formation of energy for the survival of the plant. It also plays significant roles in the responses to abiotic stress and phosphorelay signaling (Morigasaki et al., 2008).

### 3.8 Chilling-responsive “arginine and proline metabolism”, peroxisome and “RNA transport” phosphoproteins in the root

Phosphoproteins in the significantly enriched pathways in the root of tobacco under chilling stress were identified by extracting the differential phosphopeptide corresponding proteins from the phosphoproteome. Three differential phosphoproteins were involved in the “arginine and proline metabolism” pathway, including one N-carbamoylputrescine amidase-like and two delta-1-pyrroline-5-carboxylate synthases (P5CS), and they significantly increased and decreased in the root, respectively, at the levels of phosphopeptide abundance (**Supplementary Table S6**). N-carbamoylputrescine amidase (CPA), also known as N-carbamoylputrescine amidohydrolase, hydrolyzes N-carbamoylputrescine to putrescine (Sekula et al., 2016). This step is the final reaction in the biosynthesis of putrescine from arginine. The accumulation of putrescine is associated with chilling tolerance in plants. For example, putrescine coordinates the chilling tolerance of sweet orange (*Citrus sinensis* [L.] Osbeck) (Song et al., 2022). Proline is another compound that is associated with chilling resistance, and it is well-known for its role as an important osmotic regulator. It is primarily synthesized from the glutamate pathway under osmotic stress (Delauney and Verma, 1993). P5CS is the rate-limiting enzyme in the conversion of glutamate to proline (Hong et al., 2000). The *OsP5CS* mRNA rapidly accumulates in rice under chilling treatment (Igarashi et al., 1997), and it is followed by the accumulation of proline. In summary, the phosphorylation of CPA and dephosphorylation of P5CS were hypothesized to be positive responses to accumulate putrescine and proline in the root under chilling stress, respectively.

In this study, three differential phosphoproteins related to the peroxisome pathway were identified in the root, and they were peroxin-5 (PEX5) and peroxisomal membrane proteins PEX16 and PEX14 (**Supplementary Table S6**). Among them, PEX5 increased significantly, while PEX16 and PEX14 decreased at the levels of phosphopeptide abundance. This pathway was also significantly enriched in the rice variety MWG, which is resistant to *Magnaporthe oryzae*, the causal agent of rice blast, when the plant has been inoculated with this fungus (Peng et al., 2023). Peroxisomes are dynamic organelles that are enveloped in membranes, and they enable the cells to rapidly respond to changing conditions. They are involved in various pathways, such as the β-oxidation of fatty acids and the scavenging of reactive oxygen species (ROS) (Wang and Subramani, 2017; Gaussmann et al., 2021). Many peroxisomal proteins have been proven to be phosphorylated *in vivo* (Oeljeklaus et al., 2016). Therefore, the phosphorylation and dephosphorylation of the peroxisomal proteins in this study is a rapid response of the root to chilling stress.

There were nine differential phosphoproteins related to the RNA transport pathway in the root; four increased significantly, and five decreased at the levels of phosphopeptide abundance (**Supplementary Table S6**). This pathway was also significantly enriched in phosphoproteins that responded to chilling in *A. thaliana* and cucumber (Tan et al., 2021; Zhou et al., 2023). The transport of RNA is essential for the expression of genes and proper cellular function. Therefore, the phosphorylation and dephosphorylation of the proteins in RNA transport were hypothesized to play important roles to regulate the levels of expression of tobacco genes in response to chilling stress.

### 3.9 Analysis of the transcriptomic profiles and correlations between the transcriptome and proteome of tobacco seedlings in response to chilling stress

**Figure 5:**
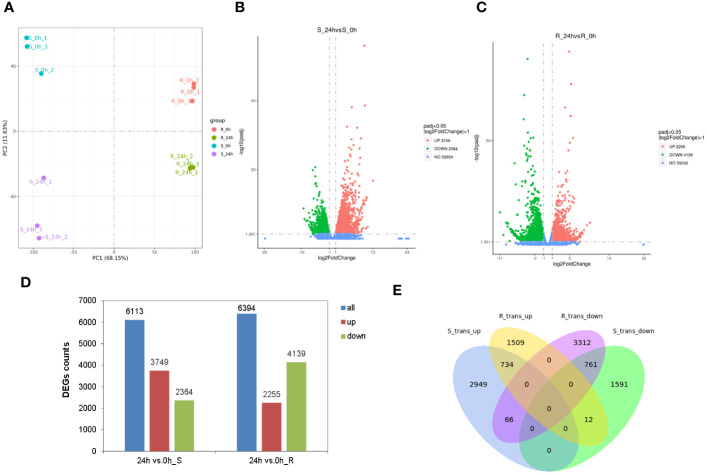
Transcriptome analysis of tobacco in response to chilling stress. **(A)** PCA plots of the transcriptome samples under chilling treatment. Volcano plots of differentially expressed genes (DEGs) in the shoot **(B)** and root **(C)**. **(D)** Comparative analysis of DEGs in the shoot and root under chilling stress. Transcripts with an adjusted *p*-value (padj) < 0.05 and |log_2_FC| > 1 were identified as DEGs. **(E)** Venn diagram of the DEGs in the shoot and root under chilling stress.

The differential transcriptional regulation of tobacco seedlings in response to chilling stress was studied by analyzing the transcriptomes of the shoot and root after 24 h of chilling treatment. The results of a Pearson correlation analysis revealed that the R^2^ values of the two groups were > 0.7 and < 0.8 among the shoot samples treated for 24 h, and the values of the other 10 groups were all > 0.8 (**Supplementary Figure S5**). This indicated that the experiments were highly reliable, and the sample selection was appropriate. The PCA showed that there was a clear separation between the chilling-treated and control samples, which indicated that the results were reproducible and reliable (**Figure 5A**). The transcriptome analysis showed that each library generated clean reads that ranged from 52.4 to 61.3 million, with the total mapped rates between 90.42% and 95.58% (**Supplementary Table S11**). A total of 81,980 genes were identified in the transcriptome, and 6,113 DEGs were identified in the shoot, including 3,749 upregulated and 2,364 downregulated (**Figures 5B, D**). In contrast, there were 6,394 DEGs in the root with 2,255 of them upregulated and 4,139 downregulated (**Figures 5C, D**). Heatmaps were generated for the 6,113 and 6,394 DEGs, respectively, as shown in **Supplementary Figure S6**. In addition, more genes were significantly upregulated in the shoot than were downregulated. However, fewer genes were significantly upregulated in the root than downregulated, which indicated the patterns of responses of the genes in the shoot and root of the tobacco seedlings varied in response to chilling stress. In addition, a qRT-PCR analysis of 10 DEGs validated the results of transcriptome and found the same patterns of expression for all the genes when they were re-examined using two independent approaches (**Supplementary Figure S7**). This indicated that the data were reliable. A Venn analysis was then performed on the DEGs in both the shoot and root, and the two tissues shared 734 upregulated and 761 downregulated genes. Additionally, there were 66 upregulated genes in the shoot that were downregulated in the root, and there were 12 downregulated genes in the shoot that were upregulated in the root (**Figure 5E**). This indicated that the patterns of expression of these genes varied in the shoot and root.

**Figure 6:**
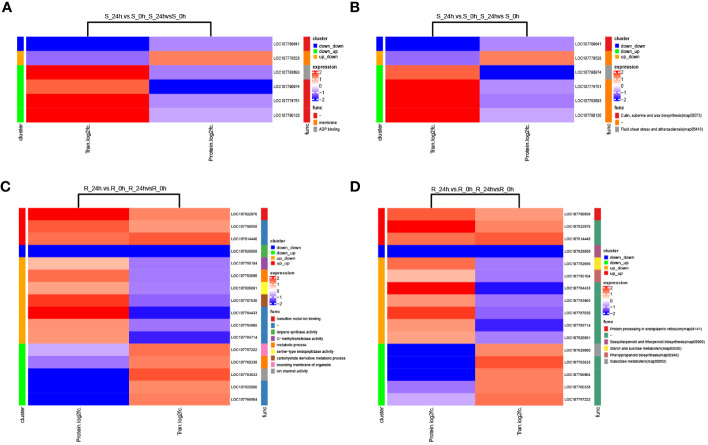
Correlation analysis of the transcriptome and proteome of tobacco under chilling stress. **(A)** GO and **(B)** KEGG enrichment analyses of pairs of DEGs/DEPs in the shoot. **(C)** GO and **(D)** KEGG enrichment analyses of pairs of DEGs/DEPs in the root.

The transcriptome and proteome data of the shoot and root were integrated to explore the association between genes and proteins, and only eight pairs of DEGs/DEPs in the shoot and 18 pairs in the root were identified (**Supplementary Figures S8A, B** **;** **Supplementary Table S12**). A correlation analysis of the fold-changes of genes (proteins) identified in both the transcriptome and proteome showed that there were weak negative correlations between the two omics of the shoot and root (**Supplementary Figures S8C, D**). The GO and KEGG enrichments were analyzed for the common pairs of DEGs/DEPs, and the eight pairs of DEGs/DEPs in the shoot were enriched to four GO terms, including membrane, ADP binding, isoprenoid biosynthetic process and integral component of membrane (**Figure 6A** **;** **Supplementary Table S12**). The 18 pairs of DEGs/DEPs in the root were enriched to 10 GO terms, including transition metal ion binding, metabolic process, and carbohydrate derivative metabolic process among others (**Figure 6C** **;** **Supplementary Table S12**). In addition, three KEGG pathways, including cutin, suberine and wax biosynthesis, glutathione metabolism and terpenoid backbone biosynthesis, were significantly enriched in the eight pairs of DEGs/DEPs (**Figure 6B** **;** **Supplementary Table S12**). Moreover, five KEGG pathways, including starch and sucrose metabolism, phenylpropanoid biosynthesis, and galactose metabolism among others, were significantly enriched in the 18 pairs of DEGs/DEPs (**Figure 6D** **;** **Supplementary Table S12**). Furthermore, common KEGG pathways that were significantly enriched in the pairs of DEGs/DEPs were compared with the ones significantly enriched in the differential phosphoproteins of the two tissues, respectively. No common pathway was identified in the shoot, while starch and sucrose metabolism was the only common one in the root. These findings suggest that this pathway plays an important role in the response of the root to chilling stress.

## 4 Discussion

Protein phosphorylation is an important post-translational modification that regulates protein function, and it plays essential roles in the mediation of chilling signal transduction in *A. thaliana*, maize and cucumber (Tan et al., 2021; Xing et al., 2022; Zhou et al., 2023). Although there has been some progress in elucidating the molecular mechanisms of the response of tobacco to chilling stress, these studies were primarily conducted at the levels of transcription (Hu et al., 2016; Luo et al., 2022) or translation (Jin et al., 2011; Hu et al., 2018). However, it is necessary to comprehensively analyze the levels of post-translational modification, particularly for the most common type of phosphorylation modification. In this study, transcriptomic, proteomic and phosphoproteomic approaches were applied to analyze the responses of tobacco shoot and root to chilling stress, thus, providing new insights to reveal the complex regulatory network of the response of tobacco to chilling stress.

### 4.1 Transcriptomic, proteomic and phosphoproteomic signatures of the tobacco seedlings in response to chilling stress

**Figure 7:**
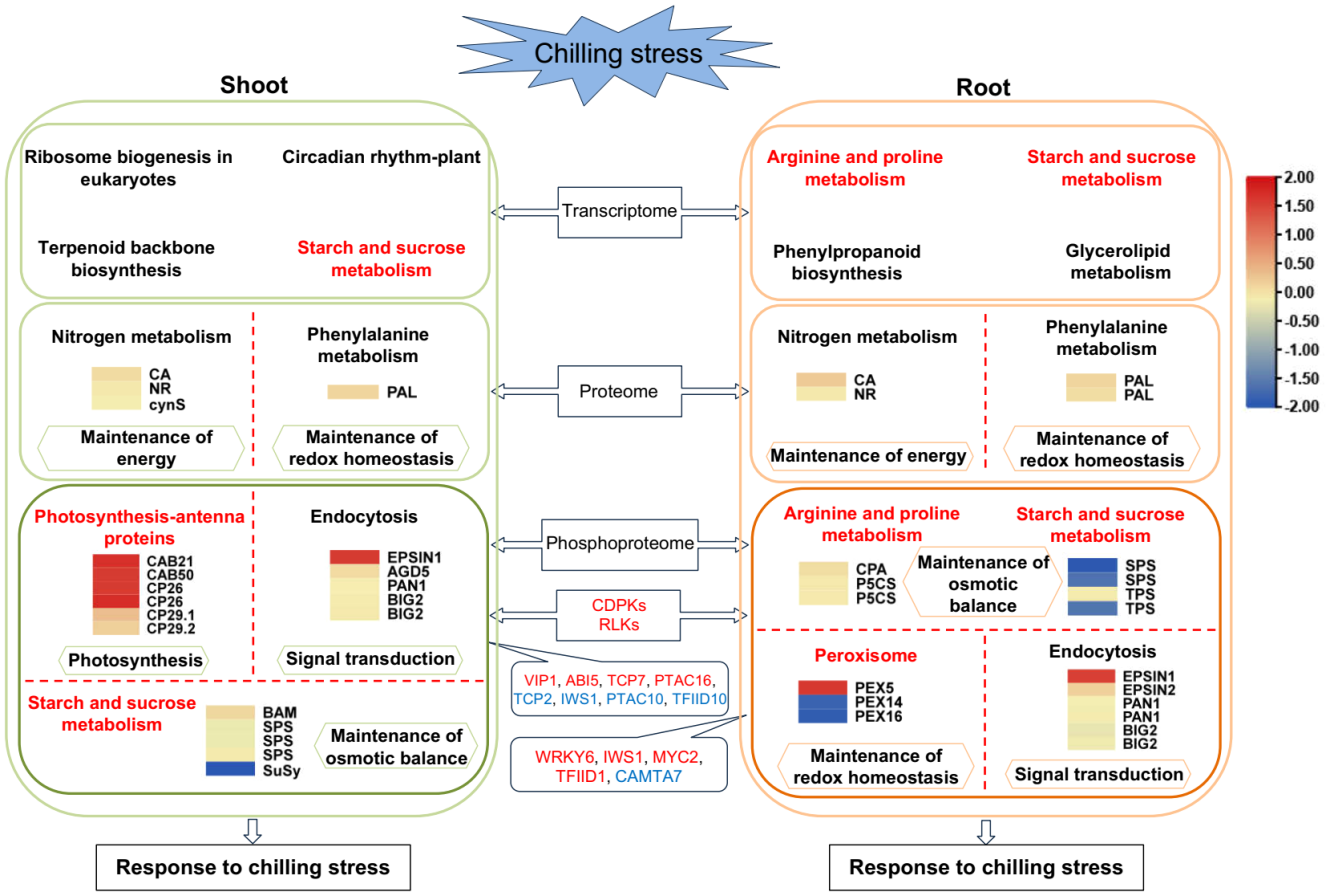
Schematic representation of a possible comprehensive chilling response model in tobacco. DEPs and differential phosphoproteins of the KEGG pathways significantly enriched in the proteome and phosphoproteome, as well as KEGG pathways that are significantly enriched in the transcriptome, are shown. The abundance of DEPs and differential phosphoproteins of the KEGG pathways was indicated with heatmaps. They were generated with scaled log_2_FC. The abundance of chilling-responsive kinases and TFs is indicated in red (increased) and blue (decreased). When tobacco seedlings were exposed to chilling stress, the chilling signal was transmitted into the cells, which triggers MAPK cascades that amplify and transmit chilling signals, activate the chilling-responsive transcription factors, induce the chilling-responsive genes, and lead to alterations in the biological processes related to chilling stress, including RNA transcription and processing, protein metabolism, photosynthesis adjustment, redox homeostasis, energy metabolism, and the accumulation of osmoregulatory compounds.

Chilling stress at the seedling stage of tobacco readily induces early flowering, which seriously affects the production and economic benefits of tobacco. It is highly significant to analyze the molecular biological mechanisms of the responses of tobacco to chilling stress. In this study, 6,113 and 6,394 DEGs, 153 and 212 DEPs, and 345 and 404 differential phosphopeptides were identified in the shoot and root under chilling stress, respectively, and there were more differential phosphoproteins than DEPs. This suggests the importance of chilling responses at the levels of phosphorylation. Among the DEGs, there were 13 and eight genes related to flowering in the shoot and root, respectively. Strikingly, they were all upregulated in the shoot, while they were upregulated and downregulated in the root (**Supplementary Table S13**). Thus, these results provide valuable marker genes for further functional characterizations. In addition, a correlation analysis of the transcriptome and proteome showed that the two omics negatively correlated, which indicated that there was a very low correlation between the two omics as previously shown (Peng et al., 2018; Mergner et al., 2020; Ye et al., 2021; Li et al., 2023b). Therefore, the significantly enriched pathways in the transcriptome (**Supplementary Table S14**) were compared with those in the phosphoproteome, and the two omics were found to share starch and sucrose metabolism in the shoot and arginine and proline metabolism, as well as starch and sucrose metabolism in the root (**Figure 7**). In addition, at the transcriptional level, the biological pathways that were significantly enriched for the DEGs were ribosome biogenesis in eukaryotes, circadian rhythm–plant and terpenoid backbone biosynthesis in the shoot, and phenylpropanoid biosynthesis and glycerolipid metabolism in the root (**Figure 7** **;** **Supplementary Table S14**). This suggested that the differential expression of the genes in these pathways is a positive response to chilling stress in tobacco seedlings. Moreover, there very few significantly different proteins were shared by the proteome and phosphoproteome, which is similar to the results of chilling response in cucumber seedlings (Zhou et al., 2023). Therefore, the pathways shared by the shoot and root were compared at the levels of protein and phosphopeptide abundance, respectively. The results of a KEGG enrichment analysis on the 153 and 212 DEPs in the shoot and root, respectively, showed that nitrogen metabolism and phenylalanine metabolism were the common pathways between the two tissues at the levels of protein abundance (**Figures 4C**, **7** **;** **Supplementary Table S4**). The results of 330 and 385 differential phosphoproteins showed that starch and sucrose metabolism and endocytosis were the common responses between the two tissues at the levels of phosphopeptide abundance (**Figures 4D**, **7** **;** **Supplementary Table S9**). Thus, these pathways may play important roles in the response of tobacco seedlings to chilling stress.

### 4.2 Phosphoproteins in the photosynthesis-antenna proteins and starch and sucrose metabolism pathways play important roles in response to chilling stress in the shoot of tobacco seedlings

Photosynthesis is particularly sensitive to temperature, particularly photosystem II (PSII). The photosystems of plants are prone to photoinhibition under chilling stress. Thus, the plants have evolved a variety of mechanisms to respond to chilling stress in which light-harvesting antenna proteins play an important role. In this study, the chlorophyll a-b binding proteins that increased significantly (**Supplementary Table S6**) were primarily CP29.1, CP29.2 and CP26 (**Figure 7**), which are trace light-harvesting antenna proteins of PSII and exist in the form of monomers. Many researchers have intensively studied CP29. For example, Bergantino et al. (1995) characterized a chlorophyll a/b binding protein that was associated with resistance to chilling stress in maize and found that it was phosphorylated CP29. The plants were more sensitive to the photoinhibition induced by chilling stress when they could not catalyze phosphorylation (Bergantino et al., 1995). Excess light can induce the phosphorylation of CP29, which was further enhanced by chilling stress, and increased the stress resistance in rice (Betterle et al., 2015). The phosphorylation of CP29 can protect the PSII reaction centers against the photoinhibition induced by the chilling stress (Mauro et al., 1997). Taken together, these studies suggest that the phosphorylation of tobacco chlorophyll a-b binding proteins could help to protect PSII under chilling stress and reduce the influence of photoinhibition. In addition, the analysis of PPI in this study showed that the hub proteins that responded to chilling stress in the shoot were primarily the photosynthesis-antenna proteins, which is consistent with the findings of the KEGG pathway enriched from the differential phosphoproteins of the shoot in this study. In addition, other plants were found to respond similarly. For example, the PPI was enriched in photosynthesis in rice, maize roots and cucumber seedlings (Sperotto et al., 2018; Ma et al., 2019; Zhou et al., 2023).

Starch and sucrose metabolism were significantly enriched in the shoot under chilling stress in this study. The soluble sugars generated in this pathway are involved in the resistance to oxidation owing to their regulation of osmotic pressure and protection of cell membrane integrity (Xu et al., 2023). This pathway plays significant roles in abiotic stresses, with the accumulation of soluble sugars as a stress signal (Xu et al., 2023). For example, sucrose was the major free sugar in response to chilling treatment in spinach (*Spinacia oleracea* L.) leaves, and the activity of SPS increased significantly (Guy et al., 1992). The activities of enzymes related to sucrose metabolism were altered at low temperatures in winter wheat, which led to an accumulation of sucrose (Zhang et al., 2019). The contents of soluble sugars, such as sucrose, trehalose, and maltose, increased during the first 12 h of chilling treatment in *Argyranthemum frutescens* (Xu et al., 2023). In this study, five differential phosphoproteins related to starch and sucrose metabolism were identified in the shoot, including BAM, SPS and SuSy (**Supplementary Table S6**). BAM increased significantly, while SPS and SuSy decreased at the levels of phosphopeptide abundance (**Figure 7**). The phosphorylation of BAM and dephosphorylation of SPS and SuSy may facilitate the accumulation of sucrose. The shoot were hypothesized to regulate osmotic pressure through the accumulation of soluble sugars that stabilize the proteins and cell structures under chilling stress.

### 4.3 Phosphoproteins in the arginine and proline metabolism, starch and sucrose metabolism, and peroxisome pathways positively respond to chilling stress in the root of tobacco seedlings

Plants under chilling, drought and salt stress contain a large amount of proline. This compound is used as an osmoregulatory agent to participate in the metabolic response to stress. Other osmoregulatory compounds, such as soluble sugars, are also important agents that protect the plants against chilling stress. In this study, three differential phosphoproteins were identified in arginine and proline metabolism, and they were N-carbamoylputrescine amidase-like and P5CS, which significantly increased and decreased in the root, respectively, at the levels of phosphopeptide abundance (**Supplementary Table S6** **;** **Figure 7**). These enzymes are important for the biosynthesis of putrescine and proline, respectively. It is well-known that putrescine is a type of polyamine that plays important roles in stabilizing membranes and macromolecules, scavenging ROS and alleviating oxidative stress, and the content of putrescine under chilling stress was elevated in sweet orange (Song et al., 2022). Similarly, proline is valuable for the detoxification of ROS and the stabilization of membranes, proteins, and subcellular structures (Rai and Penna, 2013). Its contents increased slowly and rapidly in the tobacco cultivars that were tolerant and sensitive to chilling, respectively (Hu et al., 2022).

In addition, starch and sucrose metabolism was the only common pathway of the three omics in the root in this study (**Supplementary Tables S9**, **S12**), which was also consistent with the results of enrichment in the DEGs of maize, tobacco and *Argyranthemum frutescens* under chilling treatment (Ma et al., 2019; Hu et al., 2022; Xu et al., 2023). Sucrose is well-known to increase in the chilling shock and cold hardening conditions (Newsted et al., 1991). For example, the content of sucrose increased as much as 15-fold in spinach leaves that had been acclimatized to chilling (Guy et al., 1992). Sucrose played an important role as an osmoprotectant in grape (*Vitis vinifera* L) branches to avoid cold injury (Jiang et al., 2014). In this study, four differential phosphoproteins were identified in the root, including SPS and TPS (**Supplementary Table S6** **;** **Figure 7**). They all significantly decreased at the levels of phosphopeptide abundance, and they are important for the biosynthesis of sucrose and trehalose, respectively. Sucrose and trehalose are soluble sugars that are known as osmoregulatory compounds. These soluble sugars are involved in multiple functions, such as providing resistance to oxidation, protecting cell membrane integrity, and regulating osmotic pressure (Xu et al., 2023). In summary, many soluble sugars, such as sucrose and trehalose, may accumulate in the root of tobacco seedlings, so that they can manage chilling stress.

Peroxisomes are essential organelles that may help the cells to rapidly adapt to changing conditions, and their biogenesis depends on many proteins that are known as peroxins (PEXs) (Gaussmann et al., 2021). The homeostasis of peroxisomes becomes unbalanced when the plants encounter environmental stress, such as a high concentration of ROS, and the peroxisomes conduct quality control to maintain their homeostasis by division and degradation (Wang and Subramani, 2017). Under chilling stress, plant cells accumulate ROS, which act as signal transducers to activate downstream genes and kinases (Hsu et al., 2018). In this study, three differential phosphoproteins were identified in the root, including PEX5, PEX16 and PEX14 (**Supplementary Table S6** **;** **Figure 7**). PEX5 increased significantly, while PEX16 and PEX14 decreased at the levels of phosphopeptide abundance. PEX5, PEX14 and PEX16 are all peroxins that play important roles in importing essential enzymes (Williams and Stanley, 2010), binding to PEX5 (Neuhaus et al., 2014), and facilitating the biogenesis of peroxisomes (Kim and Mullen, 2013), respectively. Taken together, these results suggest that the phosphorylation and dephosphorylation of the peroxins are positive responses to the accumulation of ROS under chilling stress.

### 4.4 Chilling-responsive CDPKs and RLKs are crucial for the signal transduction for the responses to chilling in tobacco seedlings

Conserved motifs were identified in the shoot and root to predict associated kinases. In this study, the highest ratios of pSer and pThr motifs in the shoot and root were all [sP] and [tP] (**Supplementary Table S10**), which was consistent with the findings of a previous study (van Wijk et al., 2014). [sP] and [tP] are typical proline-directed motifs in which [sP] is a potential substrate for M(A)PK, SnRK2, RLK, AGC, CDK, CDPK and SLK (van Wijk et al., 2014), and [tP] is a potential substrate of MAPK and CDK (Kamal et al., 2020). Other motifs present in large numbers that are shared by the shoot and root include [Rxxs], [sDxE], [sxD], [LxRxxs], and [sPxR] among others (**Supplementary Table S10**). Among them, [Rxxs] is another common motif that functions in various abiotic stresses in the plants that could be recognized by MAPKK and CaMK-II (van Wijk et al., 2014; Zhang et al., 2014a; Zhong et al., 2017). Motifs may be a potential substrate of the same kinase. For example, [sP] and [tP], [sDxE] and [LxRxxs], [sDxE] and [sxD], [sP] and [sPxR] are potential substrates of MAPK, CDPK, casein kinase-II (CK-II) and CDK, respectively (Schwartz and Gygi, 2005; van Wijk et al., 2014; Zhang et al., 2014b; Gao et al., 2017; Liu et al., 2019; Kamal et al., 2020). These results suggest that MAPK kinase cascades, CDPK, CK-II and CDK may play important roles in transduction of the chilling signal and regulation of the cell cycle in tobacco seedlings.

In this study, primarily CDPKs and RLKs increased significantly in both the shoot and root at the levels of phosphopeptide abundance (**Supplementary Table S5** **;** **Figure 7**). This suggests that the phosphorylation of these kinases plays an important role in the response of tobacco to chilling stress. Ca^2+^ is a universal second messenger that is involved in signal transduction in chilling stress (Tan et al., 2021). The CDPKs play an important role in the process of transmitting Ca^2+^ signals, and they participate in the responses to abiotic stresses, such as low temperature, drought and salt in the plants. For example, *VaCPK20* is involved in chilling and drought resistance in *A. thaliana* (Dubrovina et al., 2015), while *ZmCPK4*, *AtCPK6* and *OsCPK9* are involved in the resistance to drought, salt/drought and drought, respectively (Xu et al., 2010; Jiang et al., 2013; Wei et al., 2014). The five CDPKs that increased significantly in the shoot and root did not change significantly or were not found in the proteome and transcriptome in this study (**Supplementary Table S15**). RLKs are receptor-like proteins with kinase activity, which can regulate intracellular signaling through phosphorylation, and play an important role in the response to various abiotic stresses. For example, *GsLRPK* in *A. thaliana* and the FERONIA receptor-like kinase gene *MdMRLK2* in apple (*Malus domestica* L.) are involved in cold resistance (Yang et al., 2014; Jing et al., 2023). RPK1 in *A. thaliana* and the leucine-rich repeat receptor-like kinase FON1 in rice are involved in the regulation of drought and water stress, respectively (Osakabe et al., 2010; Feng et al., 2014). The *RLK* genes in rice are involved in the responses to abiotic stress and plant hormones (Gao and Xue, 2012). The five RLKs that significantly increased in the shoot and root did not change significantly or were not found in the proteome; however, two decreased, and one increased in the transcriptome in this study (**Supplementary Table S15**). These results show that tobacco seedlings primarily respond to chilling stress at the levels of phosphorylation of the CDPKs and RLKs, and this response also occurs at the RNA levels of three RLKs. Taken together, these results suggest that the phosphorylation of CDPKs and RLKs plays a crucial role in the transduction of the chilling signal.

### 4.5 Chilling-responsive TFs at the level of phosphorylation abundance are crucial for chilling resistance in tobacco seedlings

ICE–CBF–COR is the most thoroughly studied chilling response pathway (Wang et al., 2017). Studies have shown that ICE1 primarily exists in the phosphorylated form in plants under cold stress (Ding et al., 2015; Li et al., 2017; Zhang et al., 2017; Zhao et al., 2017). OST1 phosphorylates and stabilizes ICE1 in *A. thaliana*, and this promotes the expression of downstream *CBF/COR* genes under cold stress (Ding et al., 2015). However, MPK3/MPK6 also phosphorylates ICE1, which promotes its degradation and negatively regulates freezing tolerance (Li et al., 2017), whereas MPK4 constitutively suppresses the activity of MPK3/MPK6 to positively regulate the chilling response (Zhao et al., 2017). The ICE1 protein remained stable within 2 h of cold treatment, but after that it began to degrade in *A. thaliana* (Ding et al., 2015). In rice, the level of phosphorylated OsICE1 in the seedlings treated at 4 °C for 8 h was 16.3-fold higher than in the untreated ones, and similarly, it was attenuated after 8 h (Zhang et al., 2017). In this study, the ICE and CBF proteins were not found in the proteome and phosphoproteome of the shoot and root under chilling stress for 24 h. This led to the hypothesis that they may degrade with prolonged chilling treatment, while *ICE1* and *CBF* respond to chilling stress in the transcriptome. The *CBF* transcripts begin to accumulate within 15 min of exposure to cold stress (Chinnusamy et al., 2003). A total of 17 *CBF* and 4 *ICE1* genes were identified in the transcriptome. One and three *CBFs* were significantly upregulated and downregulated in the shoot, respectively, while 13 *CBFs* were significantly downregulated in the root; there were no significant changes in the four *ICE1* genes in the two tissues (**Supplementary Table S13**). Thus, the significant expression of the *CBF* and *ICE* genes in the shoot and root under chilling stress indicated that the transduction of the chilling signal in tobacco seedlings partially depends on the ICE–CBF–COR pathway.

In this study, there were several TFs that changed significantly at the levels of phosphopeptide abundance in the shoot and root, which were VIP1-like, ABI5-like protein 2, TCP7-like, WRKY 6-like, MYC2-like, PTAC16, IWS1-like, TFIID subunit 1-like, and CAMTA7 among others (**Supplementary Table S5** **;** **Figure 7**). However, most were not found in the proteome, while two significantly decreased in the transcriptome, and one increased (**Supplementary Table S15**). These results similarly showed that the response of tobacco seedlings to chilling stress primarily occurs at the phosphorylation levels of the TFs and also occurs at the RNA levels of three TFs. Among the TFs, CAMTA was reported to be involved in the response to chilling. There are six CAMTAs in *A. thaliana*, and four of them contribute to chilling responses (Tan et al., 2021). CAMTA1 and CAMTA2 function in concert with CAMTA3 to induce the levels of expression of *CBF1*, *CBF2*, and *CBF3* and other genes at 4°C, which increases the tolerance of plants to freezing (Kim et al., 2013). Notably, CAMTA1–3 and CAMTA5 are regulated by chilling stress at the phosphopeptide levels in *A. thaliana* (Tan et al., 2021). Other TFs involved in this study, such as VIP1, ABI5 and MYC2, are responsive to salt and/or drought stress (Mittal et al., 2014; Lapham et al., 2018; Verma et al., 2020; Zhao et al., 2023). WRKY6 responds to H_2_O_2_ and the stress caused by low amounts of inorganic phosphorus (Pi) and boron (Chen et al., 2012). TCP7 is a positive regulator of flowering time in *A. thaliana* (Li et al., 2021), while PTAC is essential for the expression of plastid genes in *A. thaliana* (Yu et al., 2018). IWS1 is involved in the expression of genes regulated by brassinosteroid, and TFIID is critical for stimulating the transcription of cyclin genes (Li et al., 2010; Kloet et al., 2012). Among them, VIP1 is phosphorylated by CDPK under normal conditions and dephosphorylated under hypo-osmotic stresses (Takeo and Ito, 2017). MYC2 and PTAC10 can be phosphorylated by CK-II (Yu et al., 2018; Zhu et al., 2023); IWS1 is phosphorylated by AKT (Wang et al., 2023), and the phosphorylation of PTAC16 was dependent on the light-regulated protein kinase STN7 (Ingelsson and Vener, 2012). Interestingly, these studies were related to the results of kinases predicted by the conserved motifs, and CDPK and CK-II were the kinases that were predicted in this study. In addition, STN7 is a kinase that decreased significantly in the shoot in this study (**Supplementary Table S5**). Therefore, these TFs are hypothesized to be phosphorylated by CDPK, CK-II, STN7 and other kinases, which changes their activities to finally stimulate or inhibit the expression of chilling responsive genes. Thus, they constitute a complex transcriptional regulatory network in tobacco in response to chilling stress.

## 5 Conclusion

In this study, the transcriptome, proteome and phosphoproteome were analyzed in the shoot and root of tobacco seedlings under chilling treatment to investigate the molecular mechanisms of the responses to chilling stress in tobacco seedlings. A total of 6,113 DEGs, 153 DEPs and 345 differential phosphopeptides were identified in the shoot, and 6,394, 212 and 404 in the root, respectively. This study showed that the responses of tobacco seedlings to chilling stress for 24 h were primarily at the phosphopeptide levels, and phosphoproteins in the photosynthesis-antenna proteins and starch and sucrose metabolism pathways in the shoot and the arginine and proline metabolism and peroxisome pathways in the root are crucial for the responses to chilling stress. In addition, the phosphorylation or dephosphorylation of kinases, such as CDPKs and RLKs; and TFs, such as VIP1-like, ABI5-like protein 2, TCP7-like, WRKY 6-like, MYC2-like and CAMTA7 among others; all play essential roles in the transduction of chilling signals and the transcriptional regulation of the genes that respond to chilling stress. These findings provide valuable genes, proteins and phosphoproteins to elucidate the molecular mechanisms and regulatory networks of tobacco in response to chilling stress.

## Data availability statement

The datasets presented in this study can be found in online repositories. The names of the repository/repositories and accession number(s) can be found in the article/**Supplementary Material**.

## Author contributions

XS: Formal analysis, Methodology, Visualization, Writing – original draft, Writing – review & editing. ZZ: Investigation, Validation, Writing – review & editing. FY: Formal analysis, Investigation, Writing – review & editing. YY: Formal analysis, Investigation, Writing – review & editing. JG: Formal analysis, Investigation, Writing – review & editing. JL: Formal analysis, Investigation, Writing – review & editing. TX: Investigation, Validation, Writing – review & editing. XP: Conceptualization, Funding acquisition, Project administration, Resources, Supervision, Writing – original draft, Writing – review & editing.

## References

[B1] Almadanim M. C. Alexandre B. M. Rosa M. T. G. Sapeta H. Leitão A. E. Ramalho J. C.. (2017). Rice calcium-dependent protein kinase OsCPK17 targets plasma membrane intrinsic protein and sucrose-phosphate synthase and is required for a proper cold stress response. Plant Cell Environ. 40, 1197–1213. doi: 10.1111/pce.12916 28102545

[B2] Anguenot R. Nguyen-Quoc B. Yelle S. Michaud D. (2006). Protein phosphorylation and membrane association of sucrose synthase in developing tomato fruit. Plant Physiol. Bioch. 44, 294–300. doi: 10.1016/j.plaphy.2006.06.009 16806956

[B3] Bergantino E. Dainese P. Cerovic Z. Sechi S. Bassi R. (1995). A post-translational modification of the photosystem II subunit CP29 protects maize from cold stress. J. Biol. Chem. 270, 8474–8481. doi: 10.1074/jbc.270.15.8474 7721743

[B4] Betterle N. Ballottari M. Baginsky S. Bassi R. (2015). High light-dependent phosphorylation of photosystem II inner antenna CP29 in monocots is STN7 independent and enhances nonphotochemical quenching. Plant Physiol. 167, 457–471. doi: 10.1104/pp.114.252379 25501945 PMC4326754

[B5] Chen L. G. Song Y. Li S. J. Zhang L. P. Zou C. S. Yu D. Q. (2012). The role of WRKY transcription factors in plant abiotic stresses. BBA-Gene Regul. Mech. 1819, 120–128. doi: 10.1016/j.bbagrm.2011.09.002 21964328

[B6] Chen X. Irani N. G. Friml J. (2011). Clathrin-mediated endocytosis: the gateway into plant cells. Curr. Opin. Plant Biol. 14, 674–682. doi: 10.1016/j.pbi.2011.08.006 21945181

[B7] Chinnusamy V. Ohta M. Kanrar S. Lee B. Hong X. H. Agarwal M.. (2003). ICE1: a regulator of cold-induced transcriptome and freezing tolerance in *Arabidopsis* . Gene. Dev. 17, 1043–1054. doi: 10.1101/gad.1077503 12672693 PMC196034

[B8] Chinnusamy V. Zhu J. H. Zhu J. K. (2007). Cold stress regulation of gene expression in plants. Trends Plant Sci. 12, 444–451. doi: 10.1016/j.tplants.2007.07.002 17855156

[B9] Delauney A. J. Verma D. P. S. (1993). Proline biosynthesis and osmoregulation in plants. Plant J. 4, 215–223. doi: 10.1046/j.1365-313X.1993.04020215.x

[B10] Ding Y. L. Li H. Zhang X. Y. Xie Q. Gong Z. Z. Yang S. H. (2015). OST1 kinase modulates freezing tolerance by enhancing ICE1 stability in *Arabidopsis* . Dev. Cell 32, 278–289. doi: 10.1016/j.devcel.2014.12.023 25669882

[B11] Ding Y. L. Shi Y. T. Yang S. H. (2019). Advances and challenges in uncovering cold tolerance regulatory mechanisms in plants. New Phytol. 222, 1690–1704. doi: 10.1111/nph.15696 30664232

[B12] Dubrovina A. S. Kiselev K. V. Khristenko V. S. Aleynova O. A. (2015). *VaCPK20*, a calcium-dependent protein kinase gene of wild grapevine *Vitis amurensis* Rupr., mediates cold and drought stress tolerance. J. Plant Physiol. 185, 1–12. doi: 10.1016/j.jplph.2015.05.020 26264965

[B13] Fan L. S. Li R. L. Pan J. W. Ding Z. J. Lin J. X. (2015). Endocytosis and its regulation in plants. Trends Plant Sci. 20, 388–397. doi: 10.1016/j.tplants.2015.03.014 25914086

[B14] Feng L. Gao Z. R. Xiao G. Q. Huang R. F. Zhang H. W. (2014). Leucine-rich repeat receptor-like kinase FON1 regulates drought stress and seed germination by activating the expression of ABA-responsive genes in rice. Plant Mol. Biol. Rep. 32, 1158–1168. doi: 10.1007/s11105-014-0718-0

[B15] Gao L. L. Xue H. W. (2012). Global analysis of expression profiles of rice receptor-like kinase genes. Mol. Plant 5, 143–153. doi: 10.1093/mp/ssr062 21765177

[B16] Gao J. Zhang S. He W. D. Shao X. H. Li C. Y. Wei Y. R.. (2017). Comparative phosphoproteomics reveals an important role of MKK2 in banana (*Musa* spp.) cold signal network. Sci. Rep.-UK 7, 40852. doi: 10.1038/srep40852 PMC5247763 28106078

[B17] Gaussmann S. Gopalswamy M. Eberhardt M. Reuter M. Zou P. J. Schliebs W.. (2021). Membrane interactions of the peroxisomal proteins PEX5 and PEX14. Front. Cell Dev. Biol. 9, 651449. doi: 10.3389/fcell.2021.651449 PMC8086558 33937250

[B18] Gilmour S. J. Zarka D. G. Stockinger E. J. Salazar M. P. Houghton J. M. Thomashow M. F. (1998). Low temperature regulation of the *Arabidopsis* CBF family of AP2 transcriptional activators as an early step in cold-induced *COR* gene expression. Plant J. 16, 433–442. doi: 10.1046/j.1365-313x.1998.00310.x 9881163

[B19] Gu K. Y. Hou S. Chen J. F. Guo J. G. Wang F. F. He C. G.. (2021). The physiological response of different tobacco varieties to chilling stress during the vigorous growing period. Sci. Rep.-UK 11, 22136. doi: 10.1038/s41598-021-01703-7 PMC8586257 34764409

[B20] Guo X. Y. Liu D. F. Chong K. (2018). Cold signaling in plants: insights into mechanisms and regulation. J. Integr. Plant Biol. 60, 745–756. doi: 10.1111/jipb.12706 30094919

[B21] Guy C. L. Huber J. L. A. Huber S. C. (1992). Sucrose phosphate synthase and sucrose accumulation at low temperature. Plant Physiol. 100, 502–508. doi: 10.1104/pp.100.1.502 16652990 PMC1075578

[B22] Hong Z. L. Lakkineni K. Zhang Z. M. Verma D. P. S. (2000). Removal of feedback inhibition of Δ^1^-pyrroline-5-carboxylate synthetase results in increased proline accumulation and protection of plants from osmotic stress. Plant Physiol. 122, 1129–1136. doi: 10.1104/pp.122.4.1129 10759508 PMC58947

[B23] Hsu C. C. Zhu Y. F. Arrington J. V. Paez J. S. Wang P. C. Zhu P. P.. (2018). Universal plant phosphoproteomics workflow and its application to tomato signaling in response to cold stress. Mol. Cell. Proteomics 17, 2068–2080. doi: 10.1074/mcp.TIR118.000702 30006488 PMC6166681

[B24] Hu Z. R. Yan W. J. Yang C. K. Huang X. B. Hu X. T. Li Y. Y.. (2022). Integrative analysis of transcriptome and metabolome provides insights into the underlying mechanism of cold stress response and recovery in two tobacco cultivars. Environ. Exp. Bot. 200, 104920. doi: 10.1016/j.envexpbot.2022.104920

[B25] Hu R. S. Zhu X. X. Xiang S. P. Zhan Y. G. Zhu M. D. Yin H. Q.. (2016). Comparative transcriptome analysis revealed the genotype specific cold response mechanism in tobacco. Biochem. Bioph. Res. Co. 469, 535–541. doi: 10.1016/j.bbrc.2015.12.040 26692485

[B26] Hu R. S. Zhu X. X. Xiang S. P. Zhang X. W. Liu Z. Zhu L. S.. (2018). Comparative proteomic analysis reveals differential protein and energy metabolisms from two tobacco cultivars in response to cold stress. Acta Physiol. Plant 40, 19. doi: 10.1007/s11738-017-2582-7

[B27] Igarashi Y. Yoshiba Y. Sanada Y. Yamaguchi-Shinozaki K. Wada K. Shinozaki K. (1997). Characterization of the gene for Δ^1^-pyrroline-5-carboxylate synthetase and correlation between the expression of the gene and salt tolerance in *Oryza sativa* L. Plant Mol. Biol. 33, 857–865. doi: 10.1023/A:1005702408601 9106509

[B28] Ingelsson B. Vener A. V. (2012). Phosphoproteomics of *Arabidopsis* chloroplasts reveals involvement of the STN7 kinase in phosphorylation of nucleoid protein pTAC16. FEBS Lett. 586, 1265–1271. doi: 10.1016/j.febslet.2012.03.061 22616989

[B29] Jain M. Nijhawan A. Tyagi A. K. Khurana J. P. (2006). Validation of housekeeping genes as internal control for studying gene expression in rice by quantitative real-time PCR. Biochem. Bioph. Res. Co. 345, 646–651. doi: 10.1016/j.bbrc.2006.04.140 16690022

[B30] Jia Y. X. Ding Y. L. Shi Y. T. Zhang X. Y. Gong Z. Z. Yang S. H. (2016). The *cbfs* triple mutants reveal the essential functions of *CBFs* in cold acclimation and allow the definition of CBF regulons in *Arabidopsis* . New Phytol. 212, 345–353. doi: 10.1111/nph.14088 27353960

[B31] Jiang H. Y. Li W. He B. J. Gao Y. H. Lu J. X. (2014). Sucrose metabolism in grape (*Vitis vinifera* L.) branches under low temperature during overwintering covered with soil. Plant Growth. Regul. 72, 229–238. doi: 10.1007/s10725-013-9854-z

[B32] Jiang S. S. Zhang D. Wang L. Pan J. W. Liu Y. Kong X. P.. (2013). A maize calcium-dependent protein kinase gene, *ZmCPK4*, positively regulated abscisic acid signaling and enhanced drought stress tolerance in transgenic *Arabidopsis* . Plant Physiol. Bioch. 71, 112–120. doi: 10.1016/j.plaphy.2013.07.004 23911729

[B33] Jin Y. Zhang C. H. Yang H. Yang Y. H. Huang C. J. Tian Y.. (2011). Proteomic analysis of cold stress responses in tobacco seedlings. Afr. J. Biotechnol. 10, 18991–19004. doi: 10.5897/AJB11.900

[B34] Jin J. J. Zhang H. Zhang J. F. Liu P. P. Chen X. Li Z. F.. (2017). Integrated transcriptomics and metabolomics analysis to characterize cold stress responses in *Nicotiana tabacum* . BMC Genomics 18, 496. doi: 10.1186/s12864-017-3871-7 28662642 PMC5492280

[B35] Jing Y. Y. Pei T. T. Li C. R. Wang D. N. Wang Q. Chen Y. J.. (2023). Overexpression of the FERONIA receptor kinase MdMRLK2 enhances apple cold tolerance. Plant J. 115, 236–252. doi: 10.1111/tpj.16226 37006197

[B36] Kamal M. M. Ishikawa S. Takahashi F. Suzuki K. Kamo M. Umezawa T.. (2020). Large-scale phosphoproteomic study of *Arabidopsis* membrane proteins reveals early signaling events in response to cold. Int. J. Mol. Sci. 21, 8631. doi: 10.3390/ijms21228631 33207747 PMC7696906

[B37] Kim P. K. Mullen R. T. (2013). PEX16: a multifaceted regulator of peroxisome biogenesis. Front. Physiol. 4, 241. doi: 10.3389/fphys.2013.00241 PMC3759792 24027535

[B38] Kim Y. Park S. Gilmour S. J. Thomashow M. F. (2013). Roles of CAMTA transcription factors and salicylic acid in configuring the low-temperature transcriptome and freezing tolerance of *Arabidopsis* . Plant J. 75, 364–376. doi: 10.1111/tpj.12205 23581962

[B39] Kloet S. L. Whiting J. L. Gafken P. Ranish J. Wang E. H. (2012). Phosphorylation-dependent regulation of cyclin D1 and cyclin A gene transcription by TFIID subunits TAF1 and TAF7. Mol. Cell. Biol. 32, 3358–3369. doi: 10.1128/MCB.00416-12 22711989 PMC3434555

[B40] Kolupaev Y. E. Horielova E. I. Yastreb T. O. Popov Y. V. Ryabchun N. I. (2018). Phenylalanine ammonia-lyase activity and content of flavonoid compounds in wheat seedlings at the action of hypothermia and hydrogen sulfide donor. Ukr. Biochem. J. 90, 12–20. doi: 10.15407/ubj90.06.012

[B41] Lapham R. Lee L. Y. Tsugama D. Lee S. Mengiste T. Gelvin S. B. (2018). *VIP1* and its homologs are not required for *Agrobacterium*-mediated transformation, but play a role in *Botrytis* and salt stress responses. Front. Plant Sci. 9, 749. doi: 10.3389/fpls.2018.00749 PMC6005860 29946325

[B42] Li H. Ding Y. L. Shi Y. T. Zhang X. Y. Zhang S. Q. Gong Z. Z.. (2017). MPK3- and MPK6-mediated ICE1 phosphorylation negatively regulates ICE1 stability and freezing tolerance in *Arabidopsis* . Dev. Cell 43, 630–642. doi: 10.1016/j.devcel.2017.09.025 29056553

[B43] Li Y. C. Fan K. Shen J. Z. Wang Y. Jeyaraj A. Hu S. K.. (2023a). Glycine-induced phosphorylation plays a pivotal role in energy metabolism in roots and amino acid metabolism in leaves of tea plant. Foods 12, 334. doi: 10.3390/foods12020334 36673426 PMC9858451

[B44] Li L. Ye H. X. Guo H. Q. Yin Y. H. (2010). *Arabidopsis* IWS1 interacts with transcription factor BES1 and is involved in plant steroid hormone brassinosteroid regulated gene expression. P. Natl. Acad. Sci. U.S.A. 107, 3918–3923. doi: 10.1073/pnas.0909198107 PMC2840484 20139304

[B45] Li Y. Zhang Z. P. Jiang S. H. Xu F. Tulum L. Li K. X.. (2023b). Using transcriptomics, proteomics and phosphoproteomics as new approach methodology (NAM) to define biological responses for chemical safety assessment. Chemosphere 313, 137359. doi: 10.1016/j.chemosphere.2022.137359 36427571

[B46] Li X. Y. Zhang G. F. Liang Y. H. Hu L. Zhu B. N. Qi D. M.. (2021). TCP7 interacts with Nuclear Factor-Ys to promote flowering by directly regulating *SOC1* in *Arabidopsis* . Plant J. 108, 1493–1506. doi: 10.1111/tpj.15524 34607390

[B47] Liang G. P. Hou Y. J. Wang H. Wang P. Mao J. Chen B. H. (2023). VaBAM1 weakens cold tolerance by interacting with the negative regulator VaSR1 to suppress β-amylase expression. Int. J. Biol. Macromol. 225, 1394–1404. doi: 10.1016/j.ijbiomac.2022.11.197 36436609

[B48] Liu Z. L. Li Y. J. Hou H. Y. Zhu X. C. Rai V. He X. Y.. (2013). Differences in the arbuscular mycorrhizal fungi-improved rice resistance to low temperature at two N levels: Aspects of N and C metabolism on the plant side. Plant Physiol. Bioch. 71, 87–95. doi: 10.1016/j.plaphy.2013.07.002 23896605

[B49] Liu H. Wang F. F. Peng X. J. Huang J. H. Shen S. H. (2019). Global phosphoproteomic analysis reveals the defense and response mechanisms of *Jatropha Curcas* seedling under chilling stress. Int. J. Mol. Sci. 20, 208. doi: 10.3390/ijms20010208 30626061 PMC6337099

[B50] Luo Z. Y. Zhou Z. C. Li Y. Y. Tao S. T. Hu Z. R. Yang J. S.. (2022). Transcriptome-based gene regulatory network analyses of differential cold tolerance of two tobacco cultivars. BMC Plant Biol. 22, 369. doi: 10.1186/s12870-022-03767-7 35879667 PMC9316383

[B51] Ma Y. H. Li S. Y. Lin H. Pan L. Y. Yang G. W. Lai Y. H.. (2019). Effect of cold stress on gene expression and functional pathways in maize root system. Grassl. Sci. 65, 249–256. doi: 10.1111/grs.12246

[B52] Ma N. N. Zuo Y. Q. Liang X. Q. Yin B. Wang G. D. Meng Q. W. (2013). The multiple stress-responsive transcription factor *SlNAC1* improves the chilling tolerance of tomato. Physiol. Plant 149, 474–486. doi: 10.1111/ppl.12049 23489195

[B53] Marks B. McMahon H. T. (1998). Calcium triggers calcineurin-dependent synaptic vesicle recycling in mammalian nerve terminals. Curr. Biol. 8, 740–749. doi: 10.1016/S0960-9822(98)70297-0 9651678

[B54] Mauro S. Dainese P. Lannoye R. Bassi R. (1997). Cold-resistant and cold-sensitive maize lines differ in the phosphorylation of the photosystem II subunit, CP29. Plant Physiol. 115, 171–180. doi: 10.1104/pp.115.1.171 12223798 PMC158472

[B55] McMahon H. T. Boucrot E. (2011). Molecular mechanism and physiological functions of clathrin−mediated endocytosis. Nat. Rev. Mol. Cell Biol. 12, 517–533. doi: 10.1038/nrm3151 21779028

[B56] Mehrotra S. Verma S. Kumar S. Kumari S. Mishra B. N. (2020). Transcriptional regulation and signaling of cold stress response in plants: an overview of current understanding. Environ. Exp. Bot. 180, 104243. doi: 10.1016/j.envexpbot.2020.104243

[B57] Mergner J. Frejno M. Messerer M. Lang D. Samaras P. Wilhelm M.. (2020). Proteomic and transcriptomic profiling of aerial organ development in *Arabidopsis* . Sci. Data 7, 334. doi: 10.1038/s41597-020-00678-w 33037224 PMC7547660

[B58] Mettlen M. Chen P. H. Srinivasan S. Danuser G. Schmid S. L. (2018). Regulation of clathrin-mediated endocytosis. Annu. Rev. Biochem. 87, 871–896. doi: 10.1146/annurev-biochem-062917-012644 29661000 PMC6383209

[B59] Miao Y. S. Tipakornsaowapak T. Zheng L. Z. Mu Y. G. Lewellyn E. (2018). Phospho-regulation of intrinsically disordered proteins for actin assembly and endocytosis. FEBS J. 285, 2762–2784. doi: 10.1111/febs.14493 29722136

[B60] Mittal A. Gampala S. S. L. Ritchie G. L. Payton P. Burke J. J. Rock C. D. (2014). Related to ABA-Insensitive3(ABI3)/Viviparous1 and AtABI5 transcription factor coexpression in cotton enhances drought stress adaptation. Plant Biotechnol. J. 12, 578–589. doi: 10.1111/pbi.12162 24483851 PMC4043863

[B61] Morigasaki S. Shimada K. Ikner A. Yanagida M. Shiozaki K. (2008). Glycolytic enzyme GAPDH promotes peroxide stress signaling through multistep phosphorelay to a MAPK cascade. Mol. Cell 30, 108–113. doi: 10.1016/j.molcel.2008.01.017 18406331 PMC2374695

[B62] Naramoto S. Kleine-Vehn J. Robert S. Fujimoto M. Dainobu T. Paciorek T.. (2010). ADP-ribosylation factor machinery mediates endocytosis in plant cells. P. Natl. Acad. Sci. U.S.A. 107, 21890–21895. doi: 10.1073/pnas.1016260107 PMC3003034 21118984

[B63] Narasimhan M. Johnson A. Prizak R. Kaufmann W. A. Tan S. T. Casillas-Pérez B.. (2020). Evolutionarily unique mechanistic framework of clathrin-mediated endocytosis in plants. eLife 9, e52067. doi: 10.7554/eLife.52067 31971511 PMC7012609

[B64] Neuhaus A. Kooshapur H. Wolf J. Meyer N. H. Madl T. Saidowsky J.. (2014). A novel pex14 protein-interacting site of human pex5 is critical for matrix protein import into peroxisomes. J. Biol. Chem. 289, 437–448. doi: 10.1074/jbc.M113.499707 24235149 PMC3879566

[B65] Newsted W. J. Chibbar R. N. Georges F. (1991). Effect of low temperature stress on the expression of sucrose synthetase in spring and winter wheat plants. Development of a monoclonal antibody against wheat germ sucrose synthetase. Biochem. Cell Biol. 69, 36–41. doi: 10.1139/o91-005 1828354

[B66] Oeljeklaus S. Schummer A. Mastalski T. Platta H. W. Warscheid B. (2016). Regulation of peroxisome dynamics by phosphorylation. BBA-Mol. Cell Res. 1863, 1027–1037. doi: 10.1016/j.bbamcr.2015.12.022 26775584

[B67] Osakabe Y. Mizuno S. Tanaka H. Maruyama K. Osakabe K. Todaka D.. (2010). Overproduction of the membrane-bound receptor-like protein kinase 1, RPK1, enhances abiotic stress tolerance in *Arabidopsis* . J. Biol. Chem. 285, 9190–9201. doi: 10.1074/jbc.M109.051938 20089852 PMC2838338

[B68] Pan X. Y. Chen J. B. Yang A. G. Yuan Q. H. Zhao W. C. Xu T. Y.. (2021). Comparative transcriptome profiling reveals defense-related genes against *Ralstonia solanacearum* infection in tobacco. Front. Plant Sci. 12, 767882. doi: 10.3389/fpls.2021.767882 PMC8712766 34970284

[B69] Pappi P. Nikoloudakis N. Fanourakis D. Zambounis A. Delis C. Tsaniklidis G. (2021). Differential triggering of the phenylpropanoid biosynthetic pathway key genes transcription upon cold stress and viral infection in tomato leaves. Horticulturae 7, 448. doi: 10.3390/horticulturae7110448

[B70] Penfield S. (2008). Temperature perception and signal transduction in plants. New Phytol. 179, 615–628. doi: 10.1111/j.1469-8137.2008.02478.x 18466219

[B71] Peng Z. He S. P. Gong W. F. Xu F. F. Pan Z. E. Jia Y. H.. (2018). Integration of proteomic and transcriptomic profiles reveals multiple levels of genetic regulation of salt tolerance in cotton. BMC Plant Biol. 18, 128. doi: 10.1186/s12870-018-1350-1 29925319 PMC6011603

[B72] Peng W. Y. Wang Y. S. Zeng X. N. Li W. Song N. Liu J.. (2023). Integrative transcriptomic, proteomic, and phosphoproteomic analysis on the defense response to *Magnaporthe oryzae* reveals different expression patterns at the molecular level of durably resistant rice cultivar Mowanggu. Front. Plant Sci. 14, 1212510. doi: 10.3389/fpls.2023.1212510 PMC10373791 37521912

[B73] Polit J. T. Ciereszko I. (2012). Sucrose synthase activity and carbohydrates content in relation to phosphorylation status of *Vicia faba* root meristems during reactivation from sugar depletion. J. Plant Physiol. 169, 1597–1606. doi: 10.1016/j.jplph.2012.04.017 22770419

[B74] Rai A. N. Penna S. (2013). Molecular evolution of plant *P5CS* gene involved in proline biosynthesis. Mol. Biol. Rep. 40, 6429–6435. doi: 10.1007/s11033-013-2757-2 24068435

[B75] Schwartz D. Gygi S. P. (2005). An iterative statistical approach to the identification of protein phosphorylation motifs from large-scale data sets. Nat. Biotechnol. 23, 1391–1398. doi: 10.1038/nbt1146 16273072

[B76] Sekula B. Ruszkowski M. Malinska M. Dauter Z. (2016). Structural investigations of N-carbamoylputrescine amidohydrolase from *Medicago truncatula*: insights into the ultimate step of putrescine biosynthesis in plants. Front. Plant Sci. 7, 350. doi: 10.3389/fpls.2016.00350 PMC4812014 27066023

[B77] Shi Y. T. Ding Y. L. Yang S. H. (2018). Molecular regulation of CBF signaling in cold acclimation. Trends Plant Sci. 23, 623–637. doi: 10.1016/j.tplants.2018.04.002 29735429

[B78] Sierro N. Battey J. N. D. Ouadi S. Bakaher N. Bovet L. Willig A.. (2014). The tobacco genome sequence and its comparison with those of tomato and potato. Nat. Commun. 5, 3833. doi: 10.1038/ncomms4833 24807620 PMC4024737

[B79] Song J. Wu H. He F. Qu J. Wang Y. Li C. L.. (2022). *Citrus sinensis* CBF1 functions in cold tolerance by modulating putrescine biosynthesis through regulation of *Arginine Decarboxylase* . Plant Cell Physiol. 63, 19–29. doi: 10.1093/pcp/pcab135 34478552

[B80] Sperotto R. A. de Araújo Junior A. T. Adamski J. M. Cargnelutti D. Ricachenevsky F. K. de Oliveira B. H. N.. (2018). Deep RNAseq indicates protective mechanisms of cold-tolerant *indica* rice plants during early vegetative stage. Plant Cell Rep. 37, 347–375. doi: 10.1007/s00299-017-2234-9 29151156

[B81] Takeo K. Ito T. (2017). Subcellular localization of VIP1 is regulated by phosphorylation and 14–3-3 proteins. FEBS Lett. 591, 1972–1981. doi: 10.1002/1873-3468.12686 28542772

[B82] Tan T. C. Valova V. A. Malladi C. S. Graham M. E. Berven L. A. Jupp O. J.. (2003). Cdk5 is essential for synaptic vesicle endocytosis. Nat. Cell Biol. 5, 701–710. doi: 10.1038/ncb1020 12855954

[B83] Tan J. J. Zhou Z. J. Feng H. Q. Xing J. Y. Niu Y. J. Deng Z. P. (2021). Data-independent acquisition-based proteome and phosphoproteome profiling reveals early protein phosphorylation and dephosphorylation events in *Arabidopsis* seedlings upon cold exposure. Int. J. Mol. Sci. 22, 12856. doi: 10.3390/ijms222312856 34884660 PMC8657928

[B84] van Wijk K. J. Friso G. Walther D. Schulze W. X. (2014). Meta-analysis of *Arabidopsis thaliana* phospho-proteomics data reveals compartmentalization of phosphorylation motifs. Plant Cell 26, 2367–2389. doi: 10.1105/tpc.114.125815 24894044 PMC4114939

[B85] Verma D. Jalmi S. K. Bhagat P. K. Verma N. Sinha A. K. (2020). A bHLH transcription factor, MYC2, imparts salt intolerance by regulating proline biosynthesis in *Arabidopsis* . FEBS J. 287, 2560–2576. doi: 10.1111/febs.15157 31782895

[B86] Wagih O. Sugiyama N. Ishihama Y. Beltrao P. (2016). Uncovering phosphorylation-based specificities through functional interaction networks. Mol. Cell. Proteomics 15, 236–245. doi: 10.1074/mcp.M115.052357 26572964 PMC4762521

[B87] Wang D. Z. Jin Y. N. Ding X. H. Wang W. J. Zhai S. S. Bai L. P.. (2017). Gene regulation and signal transduction in the ICE-CBF-COR signaling pathway during cold stress in plants. Biochem. (Moscow) 82, 1103–1117. doi: 10.1134/S0006297917100030 29037131

[B88] Wang W. Subramani S. (2017). Role of PEX5 ubiquitination in maintaining peroxisome dynamics and homeostasis. Cell Cycle 16, 2037–2045. doi: 10.1080/15384101.2017.1376149 28933989 PMC5731411

[B89] Wang Y. Zhang H. J. Ferlita A. L. Sp N. Goryunova M. Sarchet P.. (2023). Phosphorylation of IWS1 by AKT maintains liposarcoma tumor heterogeneity through preservation of cancer stem cell phenotypes and mesenchymal-epithelial plasticity. Oncogenesis 12, 30. doi: 10.1038/s41389-023-00469-z 37237004 PMC10219984

[B90] Wei S. Y. Hu W. Deng X. M. Zhang Y. Y. Liu X. D. Zhao X. D.. (2014). A rice calcium-dependent protein kinase OsCPK9 positively regulates drought stress tolerance and spikelet fertility. BMC Plant Biol. 14, 133. doi: 10.1186/1471-2229-14-133 24884869 PMC4036088

[B91] Wickham H. (2016). Ggplot2: elegant graphics for data analysis (Springer: Cham, Switzerland). doi: 10.1007/978-3-319-24277-4

[B92] Williams C. P. Stanley W. A. (2010). Peroxin 5: A cycling receptor for protein translocation into peroxisomes. Int. J. Biochem. Cell B. 42, 1771–1774. doi: 10.1016/j.biocel.2010.07.004 20633695

[B93] Xing J. Y. Tan J. J. Feng H. Q. Zhou Z. J. Deng M. Luo H. B.. (2022). Integrative proteome and phosphoproteome profiling of early cold response in maize seedlings. Int. J. Mol. Sci. 23, 6493. doi: 10.3390/ijms23126493 35742945 PMC9224472

[B94] Xu G. Y. Guo W. X. Li Z. Q. Wang C. Xu Y. L. Jin J. J.. (2022). Transcriptomic insights into the regulatory networks of chilling-induced early flower in tobacco (*Nicotiana tabacum* L.). J. Plant Interact. 17, 496–506. doi: 10.1080/17429145.2022.2055175

[B95] Xu H. Y. Li J. J. Wang L. J. Li X. Y. Liu Y. Q. Wang X.. (2023). Integrated transcriptomic and metabolomics analysis reveals abscisic acid signal transduction and sugar metabolism pathways as defense responses to cold stress in *Argyranthemum frutescens* . Environ. Exp. Bot. 205, 105115. doi: 10.1016/j.envexpbot.2022.105115

[B96] Xu J. Tian Y. S. Peng R. H. Xiong A. S. Zhu B. Jin X. F.. (2010). AtCPK6, a functionally redundant and positive regulator involved in salt/drought stress tolerance in *Arabidopsis* . Planta 231, 1251–1260. doi: 10.1007/s00425-010-1122-0 20217124

[B97] Yang L. Wu K. C. Gao P. Liu X. J. Li G. P. Wu Z. J. (2014). GsLRPK, a novel cold-activated leucine-rich repeat receptor-like protein kinase from *Glycine soja*, is a positive regulator to cold stress tolerance. Plant Sci. 215–216, 19–28. doi: 10.1016/j.plantsci.2013.10.009 24388511

[B98] Ye Z. C. Yu J. Yan W. P. Zhang J. F. Yang D. M. Yao G. L.. (2021). Integrative iTRAQ-based proteomic and transcriptomic analysis reveals the accumulation patterns of key metabolites associated with oil quality during seed ripening of *Camellia oleifera* . Hortic. Res.-England 8, 157. doi: 10.1038/s41438-021-00591-2 PMC8245520 34193845

[B99] Yin Q. Qin W. Q. Zhou Z. B. Wu A. M. Deng W. Li Z. G.. (2024). Banana MaNAC1 activates secondary cell wall cellulose biosynthesis to enhance chilling resistance in fruit. Plant Biotechnol. J. 22, 413–426. doi: 10.1111/pbi.14195 37816143 PMC10826994

[B100] Yu Q. B. Zhao T. T. Ye L. S. Cheng L. Wu Y. Q. Huang C.. (2018). pTAC10, an S1-domain-containing component of the transcriptionally active chromosome complex, is essential for plastid gene expression in *Arabidopsis thaliana* and is phosphorylated by chloroplast-targeted casein kinase II. Photosynth. Res. 137, 69–83. doi: 10.1007/s11120-018-0479-y 29330702

[B101] Zhang Z. Y. Li J. H. Li F. Liu H. H. Yang W. S. Chong K.. (2017). OsMAPK3 phosphorylates OsbHLH002/OsICE1 and inhibits its ubiquitination to activate *OsTPP1* and enhances rice chilling tolerance. Dev. Cell 43, 731–743. doi: 10.1016/j.devcel.2017.11.016 29257952

[B102] Zhang M. Lv D. W. Ge P. Bian Y. W. Chen G. X. Zhu G. R.. (2014a). Phosphoproteome analysis reveals new drought response and defense mechanisms of seedling leaves in bread wheat (*Triticum aestivum* L.). J. Proteomics 109, 290–308. doi: 10.1016/j.jprot.2014.07.010 25065648

[B103] Zhang M. Ma C. Y. Lv D. W. Zhen S. M. Li X. H. Yan Y. M. (2014b). Comparative phosphoproteome analysis of the developing grains in bread wheat (*Triticum aestivum* L.) under well-watered and water-deficit conditions. J. Proteome Res. 13, 4281–4297. doi: 10.1021/pr500400t 25145454

[B104] Zhang W. J. Wang J. Q. Huang Z. L. Mi L. Xu K. F. Wu J. J.. (2019). Effects of low temperature at booting stage on sucrose metabolism and endogenous hormone contents in winter wheat spikelet. Front. Plant Sci. 10, 498. doi: 10.3389/fpls.2019.00498 PMC6482243 31057594

[B105] Zhao W. C. Huang H. Wang J. J. Wang X. Y. Xu B. Q. Yao X. H.. (2023). Jasmonic acid enhances osmotic stress responses by MYC2-mediated inhibition of *protein phosphatase 2C1* and *response regulators 26* transcription factor in tomato. Plant J. 113, 546–561. doi: 10.1111/tpj.16067 36534116

[B106] Zhao C. Z. Wang P. C. Si T. Hsu C. C. Wang L. Zayed O.. (2017). MAP kinase cascades regulate the cold response by modulating ICE1 protein stability. Dev. Cell 43, 618–629. doi: 10.1016/j.devcel.2017.09.024 29056551 PMC5716877

[B107] Zhong M. Li S. F. Huang F. L. Qiu J. H. Zhang J. Sheng Z. H.. (2017). The phosphoproteomic response of rice seedlings to cadmium stress. Int. J. Mol. Sci. 18, 2055. doi: 10.3390/ijms18102055 28953215 PMC5666737

[B108] Zhou M. D. Li Y. S. Yan Y. Gao L. H. He C. X. Wang J.. (2023). Proteome and phosphoproteome analysis of 2,4-epibrassinolide-mediated cold stress response in cucumber seedlings. Front. Plant Sci. 14, 1104036. doi: 10.3389/fpls.2023.1104036 PMC9989176 36895878

[B109] Zhu J. K. (2016). Abiotic stress signaling and responses in plants. Cell 167, 313–324. doi: 10.1016/j.cell.2016.08.029 27716505 PMC5104190

[B110] Zhu J. H. Dong C. H. Zhu J. K. (2007). Interplay between cold-responsive gene regulation, metabolism and RNA processing during plant cold acclimation. Curr. Opin. Plant Biol. 10, 290–295. doi: 10.1016/j.pbi.2007.04.010 17468037

[B111] Zhu J. Wang W. S. Yan D. W. Hong L. W. Li T. T. Gao X.. (2023). CK2 promotes jasmonic acid signaling response by phosphorylating MYC2 in *Arabidopsis* . Nucleic Acids Res. 51, 619–630. doi: 10.1093/nar/gkac1213 36546827 PMC9881174

