# Supplementary material for: Chilling stress response in tobacco seedlings: insights from transcriptome, proteome, and phosphoproteome analyses
Source: Front Plant Sci. 2024 May 28;15:1390993. doi: 10.3389/fpls.2024.1390993 (PMC11170286; doi:10.3389/fpls.2024.1390993)
Supplement: Supplementary file 1 [file DataSheet_1.pdf]

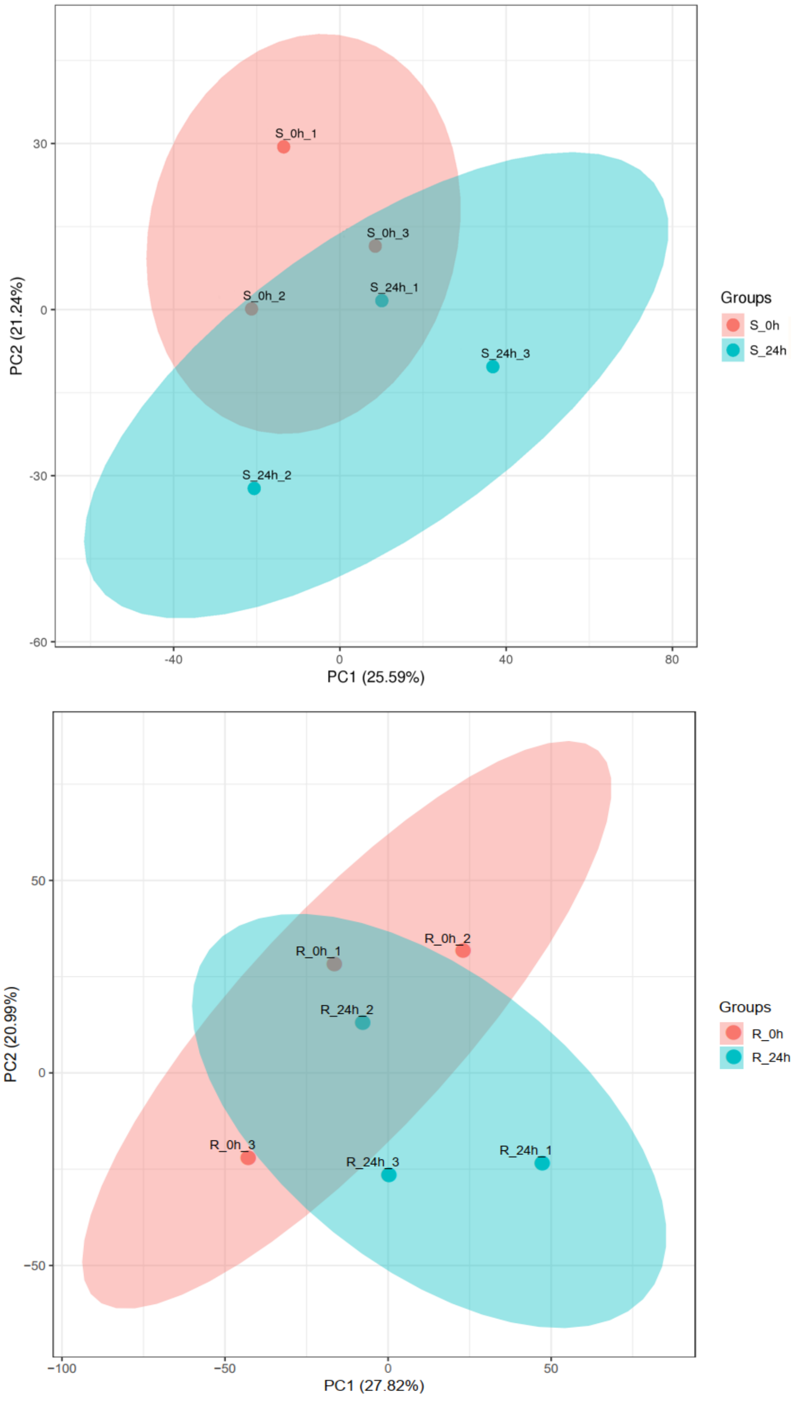

Supplementary Figure S1 PCA plots of the global proteome samples under chilling treatment. The figures above and below show the results of the shoot and root, respectively.

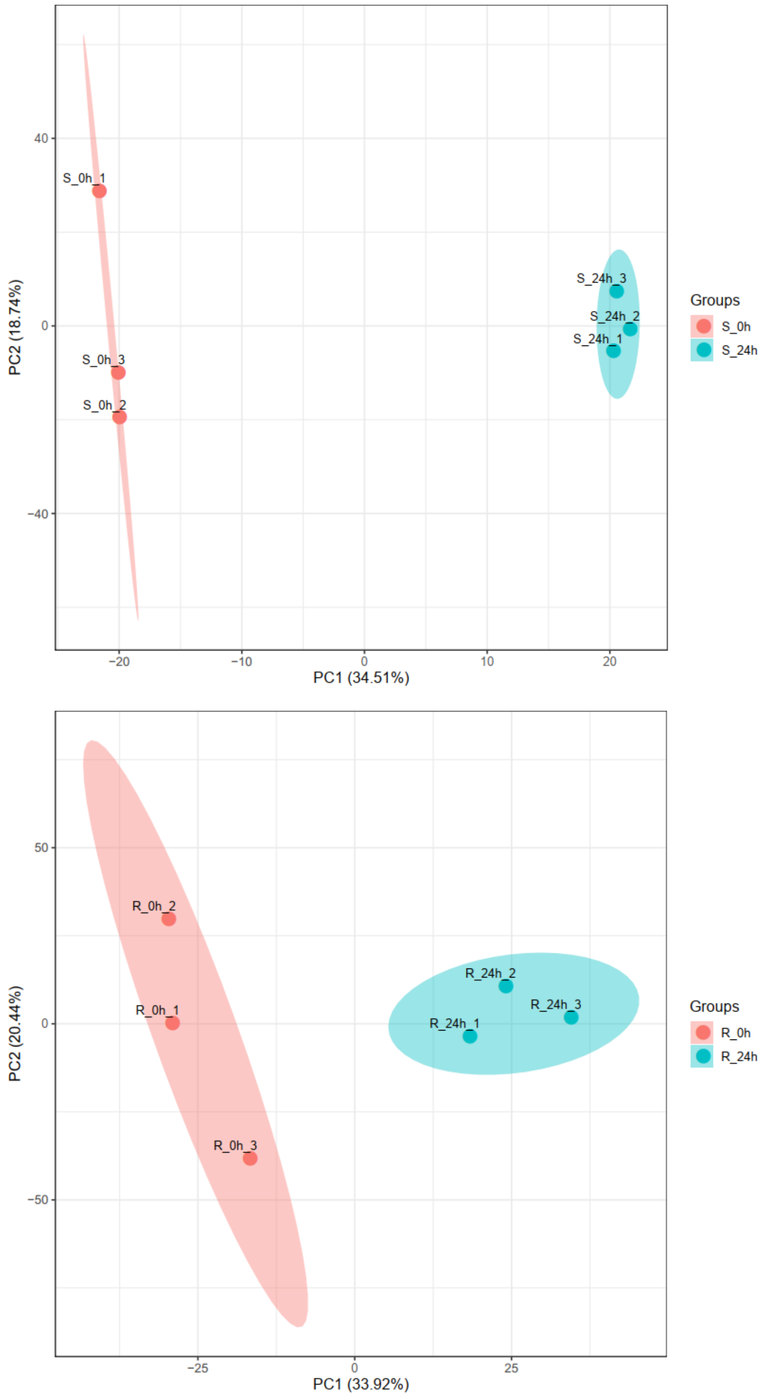

Supplementary Figure S2 PCA plots of the phosphoproteome samples under chilling treatment. The figures above and below show the results of the shoot and root, respectively.

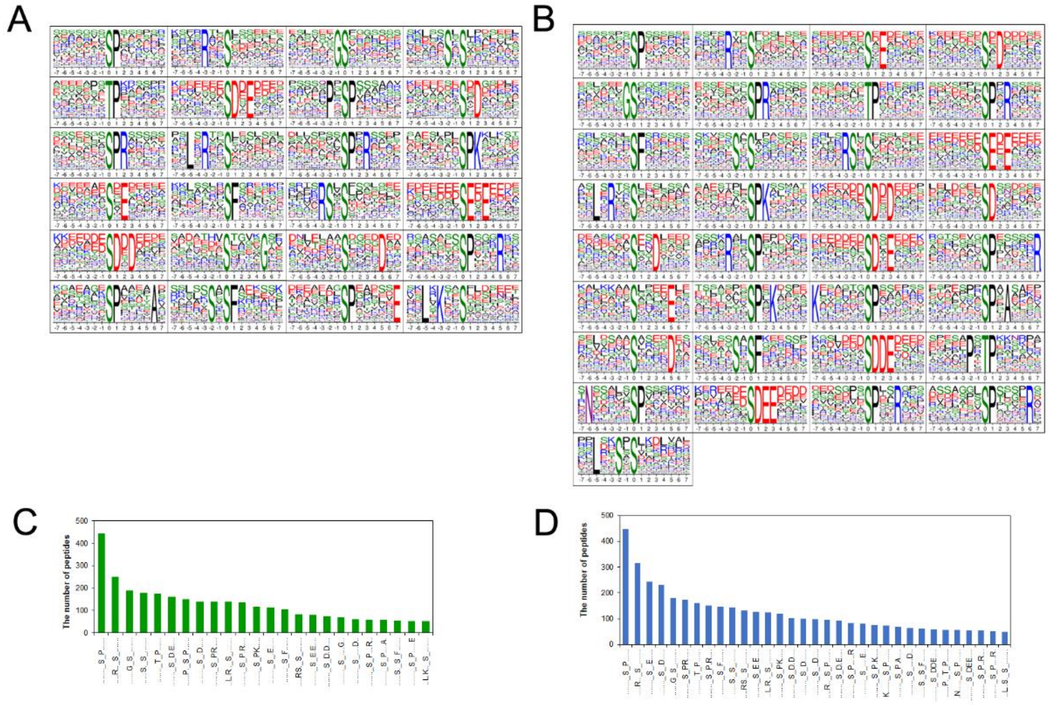

Supplementary Figure S3 Analyses of the phosphorylation sites in response to chilling stress. Sequence motif analyses of the phosphorylation sites in the shoot (A) and root (B) under chilling treatment. Number of peptides identified in the shoot (C) and root (D) that contained the phosphorylation motifs indicated. The values along the x-axis are the phosphorylation motifs that were identified.

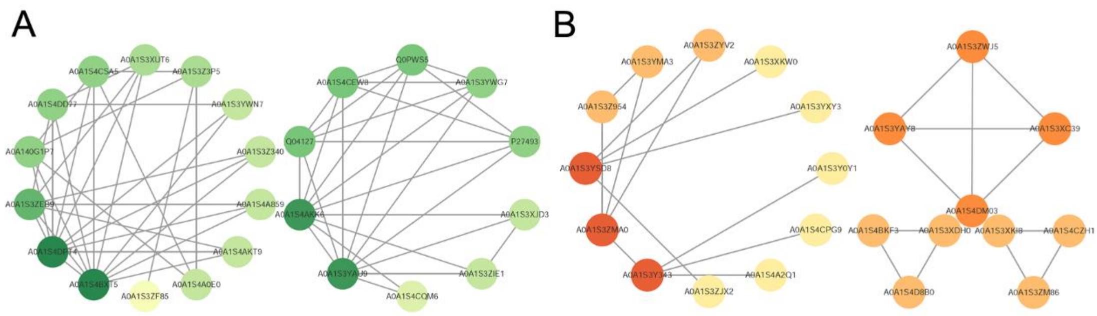

Supplementary Figure S4 The construction of PPI networks for differential phosphoproteins in the phosphoproteome under chilling stress. PPI networks of differential phosphoproteins in the shoot (A) and root (B) of tobacco seedlings under chilling stress. The interaction information of differential phosphoproteins was obtained from the shoot and root, respectively, with confidence > 0.7 as the threshold. A0A1S4DPT4, A0A1S4BXT5, A0A1S3YAU9, A0A1S4AKK6, and A0A1S3ZEB9 among others were identified as hubs in the shoot, and A0A1S3YSD8, A0A1S3ZMA0, A0A1S3XC39, and A0A1S3ZWJ5 among others were similarly identified in the root.

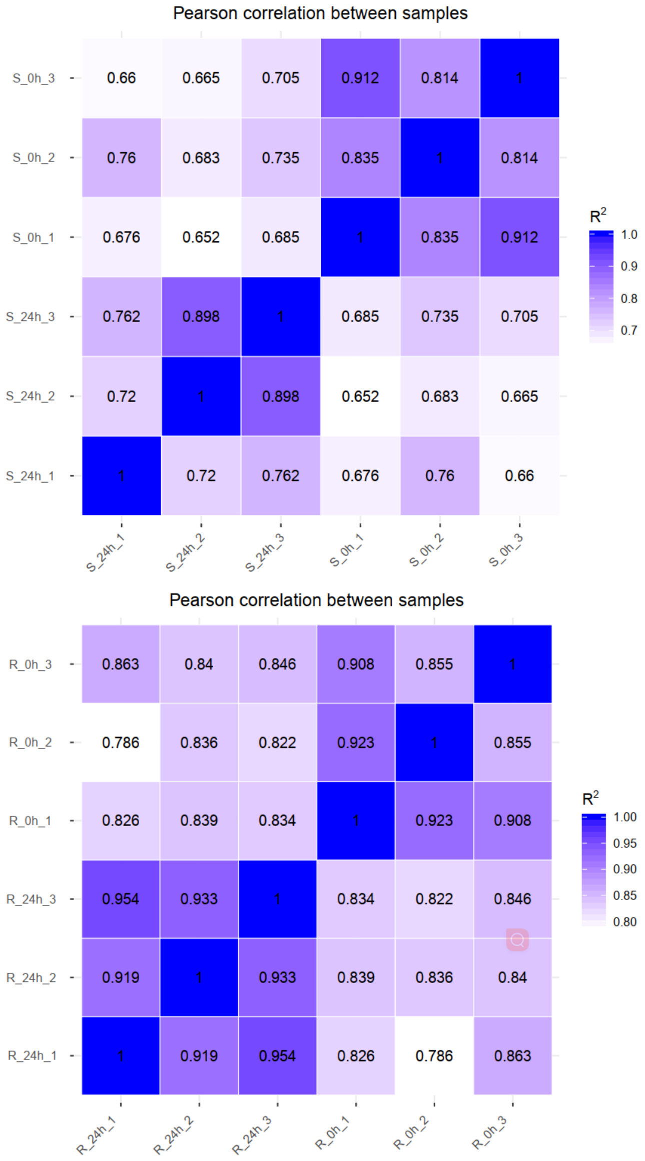

Supplementary Figure S5 Pearson correlation analysis of the transcriptome samples under chilling treatment. The figures above and below show the results of the shoot and root, respectively.

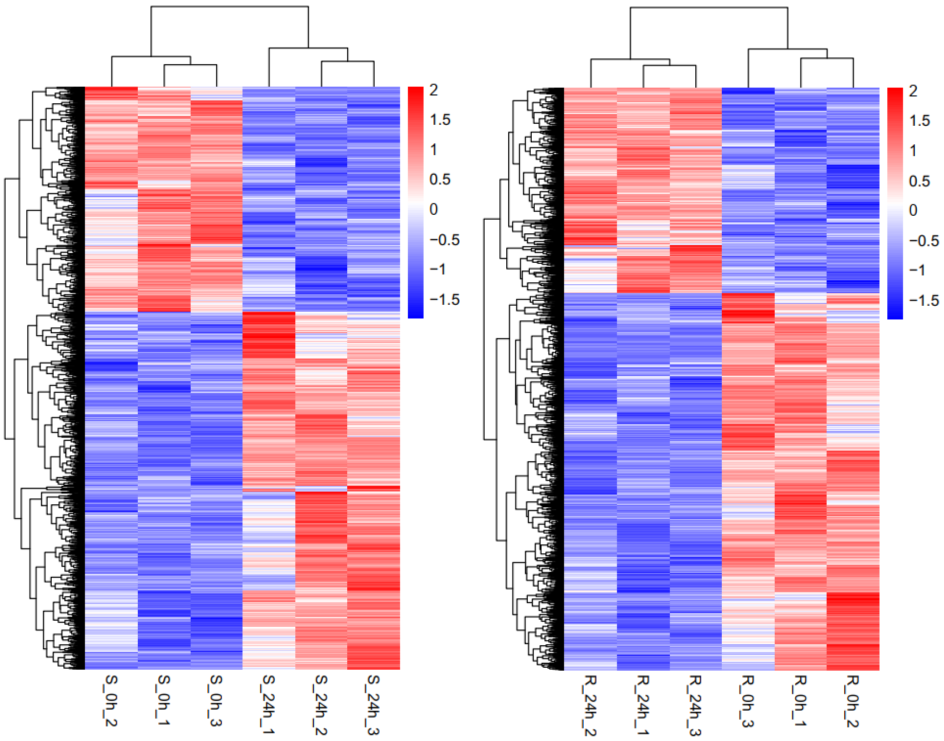

Supplementary Figure S6 Clustering heat maps of the DEGs in the transcriptome samples after chilling treatment. The left and right figures show the results of the shoot and root, respectively.

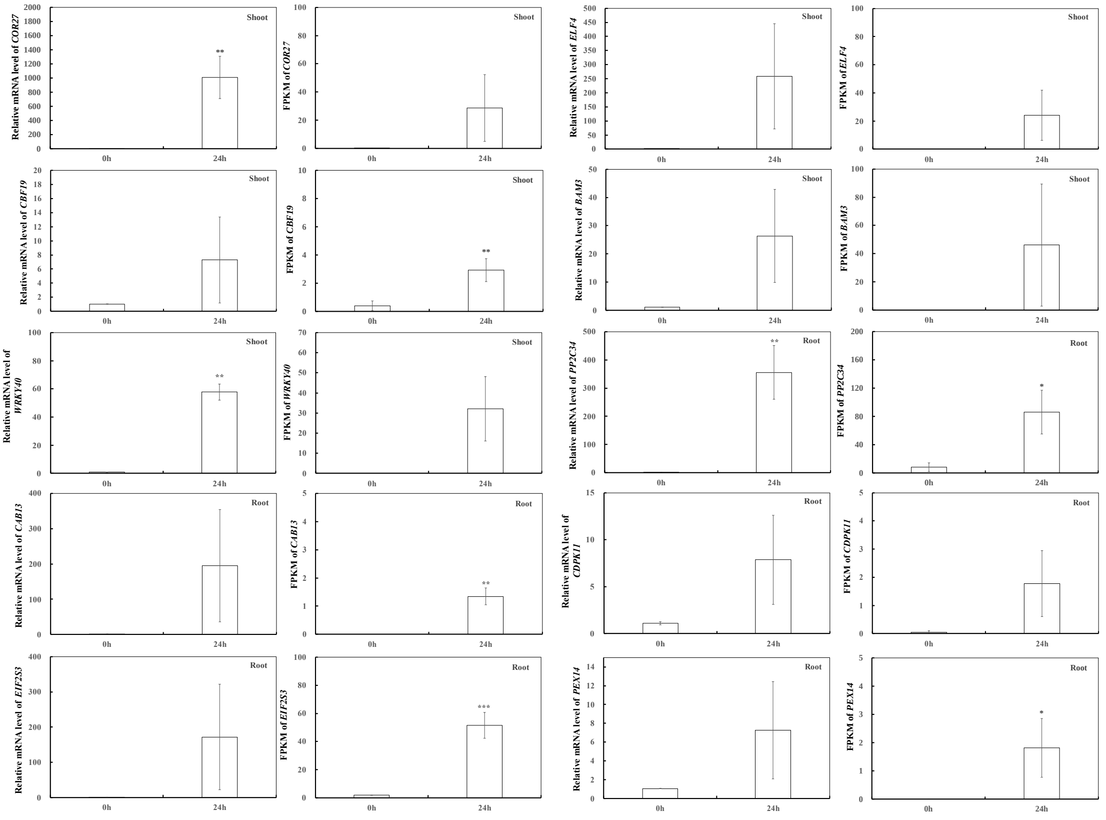

Supplementary Figure S7 qRT-PCR verification of five genes in the shoot and root under chilling treatment. The qRT-PCR data were analyzed using the $2^{-\Delta\Delta Ct}$ method. Data are the mean ± SD calculated from three biological replicates. Error bars indicate SD. *P < 0.05. **P < 0.01. ***P < 0.001. (*t*-test).

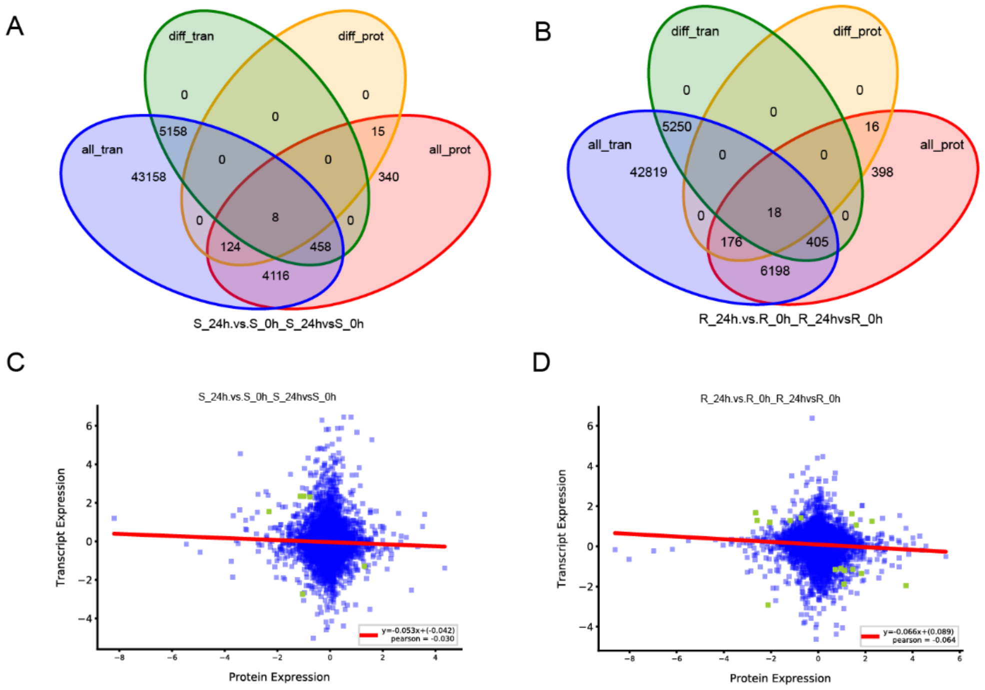

Supplementary Figure S8 Correlation analysis of the transcriptome and proteome of tobacco under chilling stress. Venn diagram (A, B) and correlation analysis (C, D) of the DEGs and DEPs between the 24 h and 0 h treatments. A and C, Venn and scatter diagram, shoot data; B and D, root data.
